# Mesenchymal stromal cells mediated delivery of photoactive nanoparticles inhibits osteosarcoma growth in vitro and in a murine in vivo ectopic model

**DOI:** 10.1186/s13046-020-01548-4

**Published:** 2020-02-22

**Authors:** Stefania Lenna, Chiara Bellotti, Serena Duchi, Elisa Martella, Marta Columbaro, Barbara Dozza, Marco Ballestri, Andrea Guerrini, Giovanna Sotgiu, Tommaso Frisoni, Luca Cevolani, Greta Varchi, Mauro Ferrari, Davide Maria Donati, Enrico Lucarelli

**Affiliations:** 1 Unit of Orthopaedic Pathology and Osteoarticular Tissue Regeneration, IRCCS Istituto Ortopedico Rizzoli, Via di Barbiano 1/10, 40136 Bologna, Italy; 2 Department of Nanomedicine, Houston Methodist Research Institute, 6670 Bertner Ave, Houston, TX 77030 USA; 3 Institute of Organic Synthesis and Photoreactivity (ISOF), National Research Council (CNR), Via Gobetti, 101, 40129 Bologna, Italy; 4 Laboratory of Musculoskeletal Cell Biology, IRCCS Istituto Ortopedico Rizzoli, Via di Barbiano 1/10, 40136 Bologna, Italy; 5 Rizzoli Laboratory Unit, Department of Biomedical and Neuromotor Sciences (DIBINEM), Alma Mater Studiorum University of Bologna, Via di Barbiano 1/10, 40123 Bologna, Italy; 6 3rd Orthopaedic and Traumatologic Clinic Prevalently Oncologic, IRCCS Istituto Ortopedico Rizzoli, Via Pupilli 1, 40136 Bologna, Italy; 7 Department of Medicine, Weill Cornell Medical College, New York, NY USA; 8 Present Address: Department of Pharmaceutics, University of Washington, Seattle, WA USA

**Keywords:** Musculoskeletal tumors, Osteosarcoma, Mesenchymal stromal cells, Photodynamic therapy, Nanoparticles, Aluminium phthalocyanine, Cell-mediated drug delivery system

## Abstract

**Background:**

Osteosarcoma (OS) is an aggressive malignant neoplasm that still suffers from poor prognosis in the case of distal metastases or occurrence of multi-drug resistance. It is therefore crucial to find novel therapeutic options able to go beyond these limitations and improve patients’ survival. The objective of this study is to exploit the intrinsic properties of mesenchymal stromal cells (MSCs) to migrate and infiltrate the tumor stroma to specifically deliver therapeutic agents directly to cancer cells. In particular, we aimed to test the efficacy of the photoactivation of MSCs loaded with nanoparticles in vitro and in a murine in vivo ectopic osteosarcoma model.

**Methods:**

AlPcS_4_@FNPs were produced by adding tetra-sulfonated aluminum phthalocyanine (AlPcS_4_) to an aqueous solution of positively charged poly-methyl methacrylate core-shell fluorescent nanoparticles (FNPs). The photodynamic therapy (PDT) effect is achieved by activation of the photosensitizer AlPcS_4_ in the near-infrared light with an LED source. Human MSCs were isolated from the bone marrow of five donors to account for inter-patients variability and used in this study after being evaluated for their clonogenicity, multipotency and immunophenotypic profile. MSC lines were then tested for the ability to internalize and retain the nanoparticles, along with their migratory properties in vitro. Photoactivation effect was evaluated both in a monolayer (2D) co-culture of AlPcS_4_@FNPs loaded MSCs with human OS cells (SaOS-2) and in tridimensional (3D) multicellular spheroids (AlPcS_4_@FNPs loaded MSCs with human OS cells, MG-63). Cell death was assessed by AnnexinV/PI and Live&Dead CalceinAM/EthD staining in 2D, while in the 3D co-culture, the cell killing effect was measured through ATP content, CalceinAM/EthD staining and TEM imaging. We also evaluated the effectiveness of AlPcS_4_@FNPs loaded MSCs as delivery systems and the ability of the photodynamic treatment to kill cancer cells in a subcutaneous mouse model of OS by bioluminescence imaging (BLI) and histology.

**Results:**

MSCs internalized AlPcS_4_@FNPs without losing or altering their motility and viability in vitro. Photoactivation of AlPcS_4_@FNPs loaded MSCs induced high level of OS cells death in the 2D co-culture. Similarly, in the 3D co-culture (MSCs:OS ratios 1:1 or 1:3), a substantial decrease of both MSCs and OS cells viability was observed. Notably, when increasing the MSCs:OS ratio to 1:7, photoactivation still caused more than 40% cells death. When tested in an in vivo ectopic OS model, AlPcS4@FNPs loaded MSCs were able to decrease OS growth by 68% after two cycles of photoactivation.

**Conclusions:**

Our findings demonstrate that MSCs can deliver functional photosensitizer-decorated nanoparticles in vitro and in vivo and inhibit OS tumor growth. MSCs may be an effective platform for the targeted delivery of therapeutic nanodrugs in a clinical scenario, alone or in combination with other osteosarcoma treatment modalities.

## Background

Osteosarcoma (OS) is a malignant aggressive primary bone tumor that commonly arises in the long bones of children and young adults. Conventional clinical treatment consists of surgical resection of the tumor and adjuvant chemotherapy [1]. In spite of the effort made by clinicians in the last 30 years, the success of OS treatment is limited to a 70% 5-years survival rate with the remaining 30% of OS patients not responding to standard treatments [2] mainly because of the formation of lung metastases, which ultimately represent the primary cause of mortality [3]. Despite testing different drugs and regimens, a substantial improvement of survival rate has not been observed [4–6]. In addition to lung metastases, OS cells resistance to chemotherapeutics, such as doxorubicin [7], greatly hampers treatments’ efficacy, therefore the development of innovative and more selective strategies capable of improving the survival of OS patients is required.

Mesenchymal stromal cells (MSCs) have been proved to be powerful tools in cell therapy, being used for a wide array of clinical indications spanning from the treatment of graft-versus-host-disease to tissue engineering, and currently are tested in several hundreds of clinical trials [8]. Additionally, thanks to their proven ability to migrate and engraft in the stroma of several tumors [9], MSCs have been used in preclinical and clinical studies as carriers of antitumor drugs with the aim of enhancing their selective accumulation at the tumor site. In 2002, Studeny et al. firstly proposed MSCs as carrier cells for gene therapy [10]; at present, several studies have been published reporting MSCs as effective delivery vehicles of anticancer agents, such as pro-apoptotic molecules, chemotherapeutic drugs and oncolytic viruses [11–20]. In addition, it has been extensively demonstrated that MSCs can internalize and deliver nanoparticles loaded with therapeutic agents [21–25], including chemotherapeutic drugs and photosensitizers (PS) for photodynamic therapy (PDT) applications [26–28].

In PDT, light at a specific wavelength is applied to the tumor site where the PS localizes after administration; once irradiated, the PS enters an excited state that triggers the formation of various reactive oxygen species (ROS) responsible for killing cancer cells and the damage of tumor vasculature that in turn deprives the tumor of oxygen and nutrients [29]. In particular, the use of nanoparticles as PS delivery systems has been proposed as treatment for several tumors with the aim of bypassing biological barriers and cellular chemo-resistance [30]. PDT has proved to be a successful, clinically approved, and minimally invasive alternative/co-adjuvant therapeutic option to conventional therapies for the treatment of various tumors [31, 32]. In particular, PDT has been proved to be effective in decreasing tumor growth both in in vitro and in vivo OS models [33–40], and in a murine model of aggressive prostate tumor [41] as well as in clinical settings [42].

In order to establish whether the PDT driven MSCs strategy is an effective system for the treatment of OS, we designed a multistep process that could allow us to determine the best operating settings, such as: doses, radiant exposure and distance of light source, time after infusion of loaded MSCs, safety of the operating procedure etc., with in mind the possibility of clinical translation. With this in mind, in an earlier study we demonstrated that MSCs can be efficiently and safely loaded with fluorescently labelled poly-methyl methacrylate nanoparticles (FNPs) electrostatically decorated with the photosensitizer tetra-phenyl sulfonated porphyrin (TPPS), and that this system (TTPS@FNP@MSCs) exerts a ROS-mediated cytotoxic effect on surrounding OS cells upon irradiation with a 405 nm light in-vitro [26]. Based on these encouraging results, we improved our NPs system by loading a different PS, i.e. tetra-sulfonated aluminum phthalocyanine (AlPcS_4_), since it is well-established that the optimal light therapeutic window, ensuring the highest tissues penetration, falls in the near-infrared region [43]. In fact, unlike TPPS, AlPcS_4_ has a strong absorption peak in the near-infrared region of the spectrum; this upgraded system, i.e. AlPcS_4_@FNPs, was able to effectively kill human prostate cancer cells in in vitro 3D model as well as in in vivo mouse model [41].

Therefore, the objective of the current study is to prove whether our AlPcS_4_@FNPs particles are an effective PDT system against OS cells; more importantly, we aim to investigate in an in vivo OS ectopic model whether AlPcS_4_@FNPs loaded into MSCs have an improved tumor selectivity compared to AlPcS_4_@FNPs alone, while maintaining their cancer cells killing efficacy.

## Material and methods

### Reagents

Dulbecco’s Modified Eagle’s Medium-high glucose (DMEM-HG, glucose 4500 mg/L), McCoy’s medium, and Ficoll®-Paque PREMIUM 1.073 reagents were purchased by Sigma Aldrich (Saint Luis, Missouri, USA). α-Minimum Essential Medium Eagle (α-MEM) was purchased by Lonza (Verviers, Belgium). Fetal Bovine Serum (FBS), GlutaMAX, penicillin/streptomycin solution, Dulbecco’s phosphate buffered solution without calcium and magnesium (D-PBS), Puromycin, Alexa Fluor® 488 Annexin V/PI Dead Cell Apoptosis Kit, LIVE/DEAD® Viability/Cytotoxicity Kit (Calcein-AM and Ethidium homodimer-1), Alamar Blue, and WST-1 assay reagents were purchased from Thermo Fisher Scientific (Waltham, Massachusetts, USA). CellTiter-GLO® was purchased from Promega (Milano, Italy). Tetra-sulfonated aluminum phthalocyanine (AlPcS_4_) was purchased from LivChem Logistics GmbH (Frankfurt, Germany).

### Preparation of AlPcS_4_@NPs/FNPs

Poly-methyl methacrylate (PMMA) core-shell fluorescent nanoparticles (FNPs) were obtained by an emulsion-co-polymerization reaction as previously described [26, 41]. Briefly, 2-(dimethyloctyl)-ammonium ethyl-methacrylate bromide (0.52 g, 1.5 mmol) in water (50 mL) was placed into a 250 mL three-necked reactor equipped with a mechanical stirrer, a condenser, a thermometer and a nitrogen inlet. The mixture was heated to 80 °C under stirring (300 rpm), and 2-aminoethyl methacrylate hydrochloride (AEMA, 0.25 g, 1.48 mmol) was added to the solution. Afterward, a mixture of allyl 2-(3-allyloxy-6-oxo-6H-xanthen-9-yl) benzoate [44] (0.003 g, 0.007 mmol) and methyl methacrylate (0.93 mL, 9.35 mmol) was added to the previously obtained solution. After 10 min, 15 mg (0.05 mmol) of 2,2′-azobis (2-methylpropionamidine) dihydrochloride (AIBA), dissolved in 0.5 mL of mQ water was added to the mixture, which was then allowed to react for 4 h. The reaction product was purified by dialysis (against water) to remove residual monomer and stabilizer. Whenever needed, and to avoid interference with fluorescent staining, non-fluorescent PMMA nanoparticles (NPs) were prepared by the same procedure without the addition of the fluorescent comonomer, i.e. allyl 2-(3-allyloxy-6-oxo-6H-xanthen-9-yl) benzoate AlPcS_4_@FNPs or AlPcS_4_@NPs stock solution was prepared by adding 50 μl of AlPcS_4_ (1 mg/mL in milliQ water) to 50 μl of FNPs or NPs (10 mg/mL) and milliQ water to a final 1 mL volume. The stock solution was diluted in complete cell culture medium to the desired concentration. Where not explicitly stated, the indicated concentrations refer to the amount of FNPs/NPs per volume unit, resulting in an equivalent AlPcS_4_ concentration of 1/10 (e.g. 90 μg/ml AlPcS_4_@FNPs are equivalent to 9 μg/mL AlPcS_4_).

### Human osteosarcoma cell lines

Human osteosarcoma cell lines, MG-63 (CRL-1427), Saos-2 (HTB-85) and U-2 OS (HTB-96) were purchased from ATCC (Manassas, Virginia, USA). Briefly, cells were cultured respectively in DMEM-HG (MG-63) or McCoy’s medium (Saos-2, U-2 OS) containing 10% of FBS, 1% GlutaMAX and 50 U/ml penicillin/streptomycin at 37 °C in a humidified atmosphere with 5% CO_2_.

Saos-2-Luc/GFP cell line was generated by transduction with Lentivirus particles containing the CMV promoter for the expression of humanized firefly luciferase (hLUC) and SV40 promoter for the expression of GFP protein according to manufacturer’s protocol (GeneCopoeia). Three days after infection, cells with high levels of GFP expression were selected by Cell Sorter NIR Aria II (BD Bioscience) and expanded for a week in culture medium supplied with Puromycin to generate a stable cell line.

### Isolation and culture of human mesenchymal stromal cells (MSCs)

MSCs were obtained from bone marrow samples of five patients undergoing surgery at Rizzoli Orthopedic Institute (Bologna, Italy). Isolation and culture expansion of human MSCs was performed as previously described in Pierini et al. [45] with minor modifications. Briefly, mononucleated cells were isolated from bone marrow through gradient separation with Ficoll®-Paque PREMIUM 1.073, then placed in 150 cm^2^ culture flasks in complete growth medium (αMEM+ 20% FBS) at a density of 4 × 10^5^ cells/cm^2^ and incubated at 37 °C in 5% CO_2_ atmosphere. Medium was changed every 3–4 days; after the first passage sub-culturing was performed at 2 × 10^3^ cell/cm^2^ every time the cells reached a 70–80% confluence. Complete characterization in terms of fibroblast-colony forming unit (CFU-F) efficiency, immunophenotypic profile, proliferation rate, and trilineage-differentiation potential of each MSC line was performed. Since ex-vivo expanded MSCs are a heterogeneous population and are known to be highly sensitive to the protocols used to isolate and expand the cells in culture [46, 47], detailed protocols are provided as Supplementary Methods (Additional file 1), and all MSCs’ characterization and in-process data are provided for review in Table 1S (Additional file 2), as suggested by Reger and Prockop [48]. In order to account for unpredictable variations on test results due to the well-known donor-to-donor variability of MSCs [49], at least 3 different MSCs lines were tested in independent experiments. When stated, all the 5 MSCs lines were tested, in order to strengthen the reproducibility of the results. Only cells from the third to the sixth passage were used in all experiments.

### Cytotoxicity assay

MSCs were seeded in 96 well plates and incubated for 1 h with AlPcS_4_@FNPs, FNPs or AlPcS_4_ at increasing doses (45, 90, 180 μg/mL for AlPcS_4_@FNPs or 4.5, 9, 18 μg/mL for AlPcS_4_). Cells were then washed twice with D-PBS and new complete medium was added to each well. WST-1 assay was performed 1, 2 and 6 days after loading following the manufacturer’s instructions. The optical density of each well was measured by a microplate reader (Synergy HT, BioTek Winooski, VT, USA) set at 450 nm with the correction wavelength set at 690 nm.

### MSCs loading with nanoparticles

MSCs were seeded at 10^4^ cells/cm^2^ in complete medium and allowed to adhere to the plates overnight before loading. MSCs were exposed to AlPcS_4_@FNPs or AlPcS_4_@NPs diluted in complete medium for 1 h, then washed two times with D-PBS. AlPcS4@FNPs or AlPcS4@NPs loaded MSCs were allowed to recover for a time varying from 2 h to overnight (o/n) in complete medium, before being detached from culture flasks, for subsequent experiments. Before each experiment, AlPcS4@FNPs loaded MSCs were checked through automated cell counter Countess II® FL (Thermo Scientific, Waltham, Massachusetts, USA) in order to verify the loading efficiency.

### Cellular uptake and accumulation

The percentage of loaded MSCs were determined by BD FACScanto II cytometer (Becton-Dickinson, Franklin Lakes, NJ, USA) and by the automated cell counter Countess II® FL (Thermo Scientific, Waltham, Massachusetts, USA), taking advantage of FITC fluorescence of FNPs. For confocal microscopy (Nikon, Amsterdam, Netherlands) analysis, MSCs were seeded onto glass coverslips, loaded with FNPs and at the indicated time points fixed for 10 min in 10% Neutral Buffered Formalin at room temperature, thoroughly washed with D-PBS, stained with Hoechst and imaged.

### In vitro migration study

Cell migration was assessed by the Boyden chamber technique. Cell culture inserts for 24-well plate with 8 μm pore diameter were used (Millipore, Darmstadt, Germany). After a recovery period of 2 h in complete medium, AlPcS_4_@FNPs loaded MSCs, along with unloaded MSCs used as control, were exposed to o/n starvation, switching the complete medium to αMEM+ 0.2%FBS. Then 10^4^ cells were placed in the upper chamber in 200 μL of αMEM+ 0.2%BSA. Six hundred microliters of αMEM supplemented with 20% FBS (chemo-attractant) or 0.2% BSA (neutral) was added into the lower chamber. After overnight incubation, cells on the upper face of the membrane were removed with a cotton swab while the ones on the bottom surface were fixed in 100% methanol and stained with Hema-stain kit (Fischer Scientific, Hampton, New Hampshire, USA). Cells migrated through the microporous membrane were counted in 10 randomly chosen fields under an inverted Nikon Eclipse TE2000-U microscope (Nikon, Amsterdam, The Netherlands).

### 2D co-culture

MSCs were loaded with 90 μg/ml AlPcS_4_@FNPs and left o/n to recover in complete medium. AlPcS_4_@FNPs loaded MSCs were then trypsinized, counted with Countess II® FL and 5 × 10^3^ cells were seeded into 24-well plate mixed with 5 × 10^3^ or 15 × 10^3^ Saos-2 cells, *i.e* 1:1 or 1:3 ratio respectively. Photoirradiation was delivered after overnight cell adhesion.

### 3D co-culture

MSCs were loaded with 90 μg/ml AlPcS_4_@FNPs and left for a recovery period of 4 h in complete medium. AlPcS_4_@FNPs loaded MSCs were then trypsinized, counted and mixed with MG-63 in different ratios (1:1, 1:3 and 1:7) to a final concentration of 10^5^ mixed cells/mL in DMEM-HG + 10%FBS. One hundred microliters aliquots of the suspension were dispensed in an ultra-low attachment U-bottom 96-well plate (Corning Costar, Amsterdam, The Nederlands) and allowed to aggregate for 4 days to form regularly shaped spheroids.

### Photodynamic therapy parameters

In in vitro experiments, AlPcS_4_@NPs loaded MSCs were photoactivated using a LED light source (λmax = 668 ± 3 nm) at room temperature, with the light-emitting unit placed directly under the tissue culture plates (radiant power: 140 mW). Monolayer cultures (2D) received photoactivation for 5 min, while spheroids (3D) for 10 min. Viability assays were performed, in all experiments, 24 h after PDT treatment.

In in vivo model, the tumor bearing area was irradiated for 20 min using the same LED source but with the addition of a focusing device (i.e. a cylinder of 0.6 cm diameter and 2 cm length, with a light-reflecting internal surface). The end of the focusing device was placed in close proximity to the mouse skin (Radiant power: 130 mW). Treatment was repeated twice, once a week.

### Cell viability assays

In 2D co-culture, cell death was evaluated by Alexa Fluor® 488 Annexin V/Propidium Iodide Dead Cell Apoptosis Kit according to the manufacturer’s protocol and analyzed with BD FACScanto II cytometer (Becton-Dickinson, Franklin Lakes, NJ, USA). Cell survival rate was determined by Alamar blue assay following the manufacturer’s instructions. The fluorescence of each well was measured by a microplate reader (Synergy HT, BioTek Winooski, VT, USA) with excitation/emission wavelengths of 530/590 nm. The fluorescence intensity from the samples was corrected using a cell-free control as blank.

For 3D co-culture system, cell death was evaluated through the ATP content–based assay CellTiter-Glo® 3D following the manufacturer’s protocol. Additionally, a LIVE/DEAD® staining was performed. Spheroids were incubated with 2.5 μM Calcein-AM in DMEM Phenol Red-free for 2 h, then Ethidium homodimer-1 (EthD-1) was added to a 5 μM final concentration for 10 min. Z-stacks images, for a total depth of 100-120 μm, were acquired with an A1R confocal laser scanner (Nikon, Amsterdam, The Netherlands) using Nikon Plan Apo VC 20x/0.75 NA DIC N2 objective lens and 3D rendering was performed with NIS elements software using the Alpha-blending algorithm.

### Transmission electron microscopy (TEM)

Spheroids were fixed with 2.5% glutaraldehyde in 0.1 M cacodylate pH 7.6 buffer for 1 h at room temperature. After post-fixation with 1% OsO_4_ in cacodylate buffer for 1 h, cells were dehydrated in an ethanol series and embedded in Epon resin. Semithin sections of 0.8 *μ*m were cut using an ultramicrotome and stained with toluidine blue. Ultrathin sections (70 nm) were contrasted with uranyl acetate and lead citrate and observed with a Jeol Jem-1011 transmission electron microscope (Jeol Jem, USA).

### Animal study

Eighteen female Athymic-nude mice, aged 6–8 weeks, were subcutaneously injected into the left flank with a mixture of Saos-2/Luc cells (1 × 10^6^) and MSCs (1 × 10^6^) in 50 μL of PBS/Matrigel. When tumors reached 100–150 mm^3^, approximately 2 weeks post-injection, the mice were divided into four groups: two control groups (group I and II respectively PBS and AlPcS_4_), group III AlPcS_4_@FNPs alone and group IV AlPcS_4_@FNPs loaded into MSCs. Fifty microliters of PBS, AlPcS_4_ (9 μg/mL), AlPcS_4_@FNPs (90 μg/mL) and AlPcS_4_@FNPs loaded-MSCs (1 × 10^6^) were intra-tumorally injected. The next day, the mice were exposed for 20 min to PDT. Intra-tumor injection and PDT treatment were performed weekly for 2 weeks. All animals were euthanized 1 week after the last treatment. After intra-tumor administration of test substances, the whole animal fluorescent imaging (excitation/emission wavelengths: 640/680 nm) was performed using the IVIS Lumina II (PerkinElmer, Waltham, MA) to observe AlPcS_4_@FNP nanoparticles biodistribution. The same instrumentation was used to monitor tumor growth through bioluminescence imaging (BLI). D-luciferin (GolBio, St Louis, MO) dissolved in PBS (1.5 mg luciferin/100 μL PBS) was injected intraperitoneally at a dose of 150 mg D-luciferin/kg. The BLI imaging was performed prior NPs/NPs loaded MSCs injections and after PDT treatment. Regions of interest (ROIs) were drawn within the tumor to measure average radiance (expressed as photons/s/cm^2^/sr) using Living Image® 4.2 software (Caliper Life Sciences, Hopkinton, MA).

### Histology

Tumors were collected, fixed in 4% paraformaldehyde solution, and embedded in paraffin. Samples were sectioned at a thickness of 4 μm and hematoxylin and eosin (H&E) staining was performed for a general inspection of the pathologic specimens. To evaluate the extent of tumor apoptosis and validate the BLI results, a terminal deoxynucleotidyl transferase dUTP nick end labeling (TUNEL) assay was performed with a commercial kit (Roche, Mannheim, Germany). *K*_i_-67 staining for cell proliferation was also performed. Images of tumor tissue were taken by a NIKON Upright BF&Fluorescent light microscope.

### Statistical analysis

All results were obtained from at least three independent experiments and expressed as the mean ± SD. Two-way ANOVA followed by Dunnett’s multiple comparisons test was used to determine statistical probabilities in the in vivo results. Results were considered to be statistically significant at *P*-values < 0.05. The statistical analysis was processed with GraphPad Prism 6 Software (GraphPad; San Diego, CA, USA).

## Results

### AlPcS_4_@FNPs internalization does not affect MSCs viability and migratory capacity

**Fig. 1:**
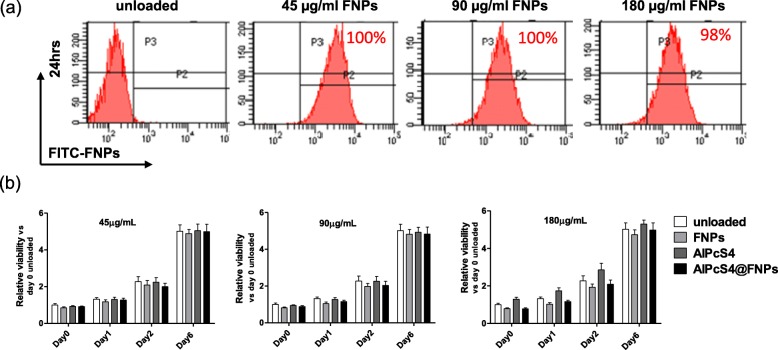
MSCs internalize AlPcS_4_@FNPs nanoparticles without cytotoxic effect. Representative flow cytometry analysis of FNPs uptake at increasing doses (45, 90, 180 μg/mL) 24 h after 1 h-loading in MSCs (**a**) and cell cytotoxicity analysis (WST-1 assay) of MSCs exposed for 1 h to increasing concentrations of FNPs, AlPcS_4_, or AlPcS_4_@FNPs, at the end of incubation (day 0) and after 1, 2 and 6 days (**b**). All data are expressed as mean ± SD (*n* = 3)

Fluorescent core-shell PMMA nanoparticles, namely FNPs, were characterized in terms of size, zeta potential, and morphology. In particular, FNPs were obtained with an average hydrodynamic diameter of 75 ± 0.92 nm (five measurements, PDI = 0.16 ± 0.01; Table 2S, Additional file 3), and a zeta-potential of 54 ± 2 mV (five measurements; Table 3S, Additional file 3). The number of ammonium group available for AlPcS_4_ loading was determined by potentiometric titration of the bromide ions obtained after complete ion exchange and was found to be 571 μmol per gram of nanospheres. The morphological analysis, performed through scanning electron microscopy (SEM), confirmed the spherical shape of FNPs (Figure 1S, Additional file 3).

In order to determine the AlPcS_4_@FNPs concentration to guarantee a 90% uptake into MSCs without altering their viability, we optimized the NPs loading parameters. Several MSC lines were incubated for 1 h with different concentrations of AlPcS_4_@FNPs (45, 90 and 180 μg/mL) and 24 h after the loading, the FITC fluorescence intensity was quantified by flow cytometry. Unloaded cells were used as control. As shown in the representative histograms of Fig. 1a, 98 to 100% of the MSCs internalized the NPs at all tested concentrations. In addition, for all concentrations, 1 h loading was sufficient for AlPcS_4_@FNPs internalization, therefore this incubation time was used in all the experiments.

To evaluate the potential cytotoxicity of AlPcS_4_@FNPs on MSCs without light irradiation, i.e. dark toxicity, cells were incubated with 45, 90 and 180 μg/mL of AlPcS_4_@FNPs for 1 h, as well as with each of the NP components alone (FNPs and AlPcS_4_). WST-1 assay was performed at 1, 2 and 6 days after loading. As shown in Fig. 1b, the viability of MSCs was not affected by the internalization of AlPcS_4_@FNPs or the single components. In fact, WST-1 values in MSCs control cells were comparable to those of MSCs exposed to AlPcS_4_@FNPs, FNPs or AlPcS_4_ at different concentrations and at all tested times (Fig. 1b).

The loading efficiency was further investigated using an automated cell counter to measure the percentage of AlPcS_4_@FNPs positive cell and the average fluorescence intensity of 5 different MSCs lines, incubated with 45, 90 and 180 μg/mL of AlPcS_4_@FNPs. Results shown in Table 1 demonstrate that, regardless of the MSC lines used, the 90 μg/mL concentration ensures the highest and most uniform internalization rate, therefore this concentration was selected for all subsequent experiments.

**Table 1:** Nanoparticles uptake tested in MSCs lines isolated from five patients

AlPcS_4_@FNPs	45 μg/mL	90 μg/mL	180 μg/mL
MSCs ID#	% positive cells	% positive cells	% positive cells
# 1	96	99	93
# 2	97	99	96
# 3	97	99	98
# 4	100	100	97
# 5	96	99	98
Average	97	99	96,4
SD.	1,6	0,4	2,07

Quantification of MSC positive cells (%) after 1 h loading with 45, 90, 180μg/mL AlPcS_4_@FNPs by Countess™ II FL

**Fig. 2:**
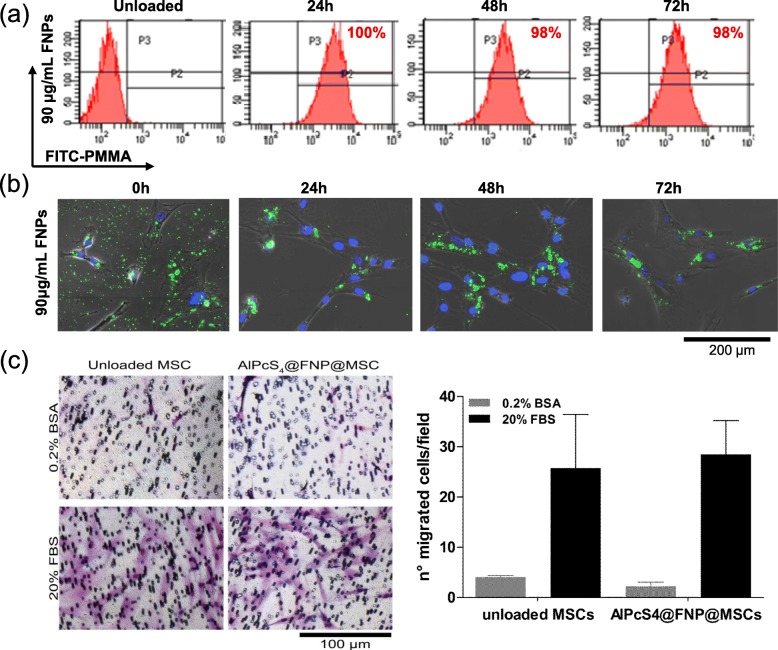
AlPcS_4_@FNPs internalization and retention analysis. Representative flow cytometry analysis of AlPcS_4_@FNPs (90 μg/mL) loaded MSCs over time (0, 24, 48, 72 h) (**a**). Representative images of FNPs internalization in MSCs after 1 h loading (0 h) over the time (up to 72 h) by confocal microscope (merge images of green (FITC of FNP) and blue (Hoechst, nuclei) channels are shown) (scale bar = 200 μm) (**b**). Representative images and quantification of cells migrated through the porous membrane of a Boyden chamber, in absence (0.2% BSA) or presence (20%FBS) of chemotactic stimuli; MSCs loaded with 90 μg/mL AlPcS_4_@FNPs were compared to unloaded MSCs (**c**). All data are expressed as mean ± SD (*n* = 3)

The retention of AlPcS_4_@FNPs (90 μg/mL) in MSCs over time (1, 2 and 3 days) was determined by flow cytometry (Fig. 2a) and by fluorescence microscopy (Fig. 2b). Both assays demonstrated that the fluorescence intensity remains constant for 3 days. In particular, the internalization was close to 100% at all tested time points, and the FNPs localization was intracellular (Fig. 2b).

Since one of the major aims of this study was to exploit MSCs as delivery vehicles of AlPcS_4_@FNPs, the migration of NPs loaded MSCs was investigated using the Boyden chamber assay. Results in Fig. 2c showed that nanoparticles internalization does not affect MSCs migration profile in both neutral and chemoattractant conditions (0.2% BSA or 20% FBS respectively), suggesting that AlPcS_4_@FNPs do not modify the migratory potential of MSCs.

### AlPcS_4_@NPs loaded MSCs induce OS cell death upon photostimulation

#### PDT efficacy in 2D co-culture system

**Fig. 3:**
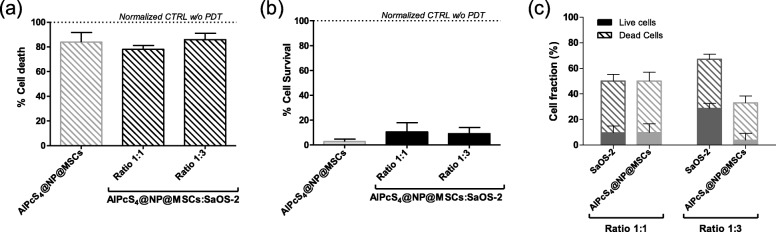
Cell death evaluation after PDT of AlPcS_4_@NPs loaded MSCs in co-culture with Saos-2 cells. Graph representing quantification of total cell death (**a**) and survival rate (**b**), 24 h after PDT by Annexin V/PI and by Alamar Blue assay respectively, 5 × 10^3^ MSCs loaded with 90 μg/ml AlPcS_4_@NPs were seeded into 24-well plate alone (grey bar) and in co-culture with 5 × 10^3^ or 15 × 10^3^ with Saos-2 cells (AlPcS_4_@NPs@MSC:Saos-2; black bars) at different ratios (1:1 and 1:3 respectively). Graph representing quantification by flow cytometry of the percentage of live or dead cells for Saos-2 (dark grey bars) and AlPcS_4_@NPs loaded MSCs (light gray bars) 24 h upon photoirradiation, 5 × 10^3^ MSCs loaded with 90 μg/ml AlPcS_4_@NPs were seeded into 24-well plate in co-culture with Saos-2 cells at 1:1 and 1:3 ratios (5 × 10^3^ or 15 × 10^3^ cells respectively) (**c**). All data are expressed as mean ± SD (*n* = 3)

To determine the effect of photoirradiation on OS cells, MSCs loaded with 90 μg/mL AlPcS_4_@NPs were co-cultured with Saos-2 cell line (ratios 1:1 and 1:3) and PDT applied for 5 min. Following irradiation, cells were kept in the dark and cultured under standard conditions for 24 h. Cell death and survival rate were evaluated by Annexin/PI labelling and Alamar Blue assay respectively; as shown in Fig. 3, nearly 80% of cell death was observed in the co-culture with Saos-2 (Fig. 3a), which was confirmed also by Alamar Blue assay that showed approximately a 20% survival rate for both the 1:1 and the 1:3 ratios (Fig. 3b). In order to distinguish the death ratio between MSCs and Saos-2 cells, AlPcS_4_@NPs loaded MSCs were co-cultured with GFP-tagged Saos-2 cells at a ratio of 1:1 and 1:3; Annexin V/PI Dead Cell Apoptosis Kit was performed. The percentage of surviving cells for each cell type was measured by flow cytometry 24 h upon photoirradiation. In particular, at the 1:1 ratio, 9% of MSCs and 12% of Saos-2 cells survived, while at the 1:3 ratio, 4% of MSCs and 28% of Saos-2 cells survived (Fig. 3c). These results demonstrate that photoirradiation is effective in killing OS cells, although the percentage of surviving OS cells is greater when the number of OS cells is increased.

#### PDT efficacy in 3D co-culture system

To further confirm the efficacy of the photoactivation of MSCs loaded with AlPcS_4_@NPs against OS cells, we developed a 3D spheroid model. Preliminary experiments were conducted using three different human OS cell lines (Saos-2, U-2 OS, MG-63), to identify the most reliable cell lines for the 3D model. MG-63 cells showed the ability to rapidly form more compact and homogeneous (in size and shape) spheroids, as respect to Saos-2 and U-2 OS (Figure 2S and Table 4S in Additional file 4) and were therefore selected for the task. Furthermore, we verified that FNPs loaded MSCs can be suitably combined with MG-63 to form multicellular spheroids, and that the FNPs were retained inside the spheroids for several days, as shown in the representative images of Figure 3Sa (Additional file 5). Indeed, from image analysis, no reduction of the fluorescence intensity due to FNPs, was observed inside the spheroids during 5 days of culture (Figure 3Sb, Additional file 5).

**Fig. 4:**
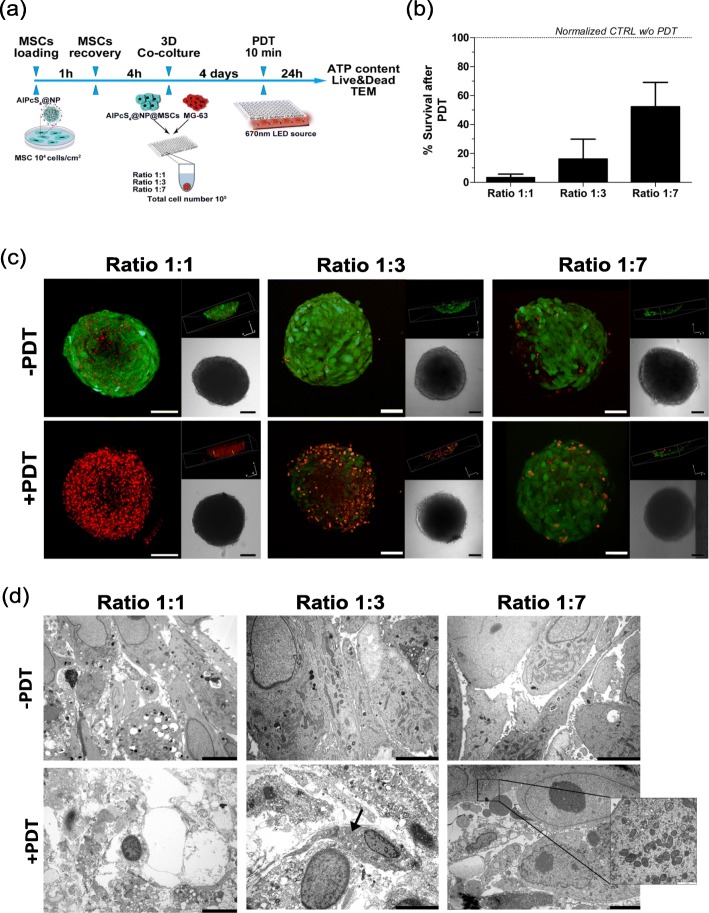
Cell death evaluation after PDT in a 3D co-culture system. Schematic summary of 3D in-vitro testing (**a**). Quantification of survival rates observed in multicellular spheroids composed by different ratios of AlPcS_4_@NPs loaded MSCs and MG-63 after 10 min irradiation. Data are expressed as mean ± SD (ratio 1:1 *n* = 5, ratio 1:3 *n* = 4, ratio 1:7 *n* = 3) (**b**). Representative confocal images (scale bar = 100 μm) of Live&Dead staining (green Calcein AM staining of live cells and red EthD-1 staining of dead cells’ nuclei) (**c**) and representative TEM images (scale bar = 5 μm) (**d**) of control (−PDT) and irradiated (+PDT) spheroids at 1:1, 1:3 and 1:7 ratios

To determine whether the efficacy of the photoactivation decreases along with the increase of cancer cells proportion, MSCs loaded with 90 μg/mL AlPcS_4_@NPs were co-cultured with MG-63 cells at different ratios, i.e. 1:1, 1:3 and 1:7 MSCs:MG-63. After 4 days from generation, spheroids were irradiated for 10 min and viability was tested after 24 h as summarized in Fig. 4a. ATP measurement showed a dramatic decrease of the cell viability at the 1:1 MSCs:MG-63 ratio (survival rate lower than 5%) in this 3D settings; additionally, all the 5 MSCs lines tested exhibited similar results as detailed in Figure 4S (Additional file 6). Moreover, when the number of MSCs in the spheroid was decreased respect to OS cells (ratios 1:3 and 1:7), a higher percentage of cell survival after 10 min of photoirradiation was observed (Fig. 4b).

Confocal images of Calcein AM/Ethidium homodimer staining showed a significant cellular death due to PDT (red cells) in the 1:1 and 1:3 ratios, while the effect was less evident in the 1:7 ratio where only few damaged cells were visible. In control spheroids (no PDT) any significant alteration of cellular viability was observed, as confirmed by the small number of dead cells found (Fig. 4c).

In order to further investigate the effect of PDT on cells, TEM analysis was performed; in particular, a high degree of cell necrosis in the whole spheroid mass of the 1:1 ratio sample was found (Fig. 4d). Similarly, the samples produced with a lower MSCs proportion showed considerable damage of the cellular structures and, even though some cellular compartments were still recognizable (Fig. 4d, black arrow), the morphology of the cytoplasm and its related organelles appeared altered. The effect of PDT treatment was less evident in the 1:7 ratio sample; in this case, besides the presence of large necrotic and altered areas, few viable cells were observed with perfectly conserved mitochondria (Fig. 4d, highlighted frame), accounting for a higher survival rate as also indicated by the ATP measurement.

**Fig. 5:**
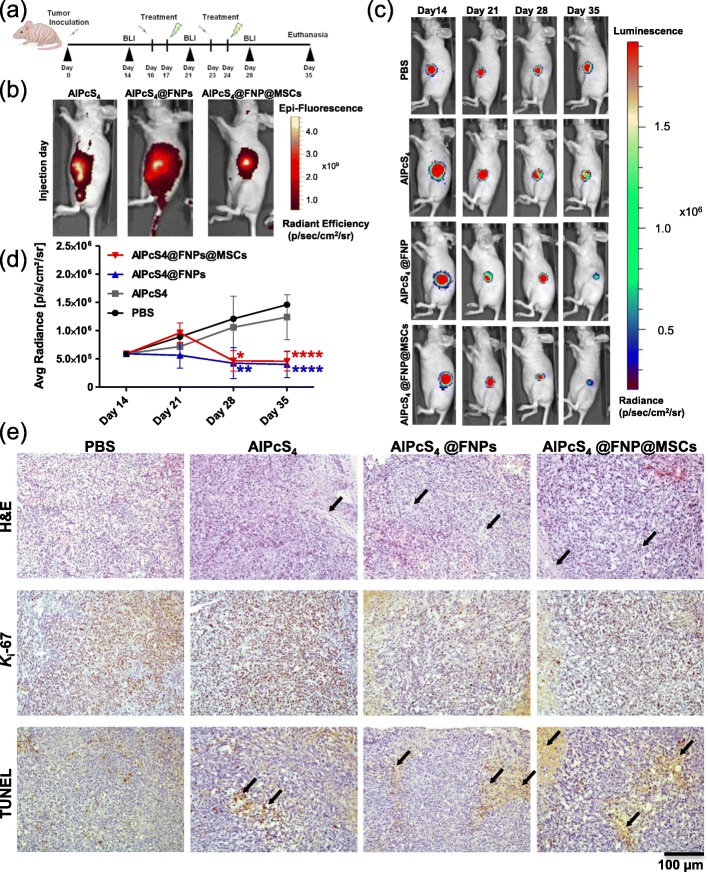
In vivo photodynamic therapy of OS tumors. Schematic representation of the in vivo treatments (**a**). Representative fluorescent luminescent imaging of AlPcS_4_ alone or loaded in NPs (AlPcS_4_@FNPs) and AlPcS_4_@FNPs loaded MSCs (AlPcS_4_@FNPs@MSCs) localization after intra-tumor injection (**b**). Representative BLI images showing the evolution of luciferase-expressing tumor cells treated (**c**). Quantification of luminescence intensity of regions-of-interest (ROI) (tumor) (the light events recorded in the acquired images expressed in mean ± SD vs time) ** *p* < 0.001 (AlPcS_4_@FNPs alone), * *p* < 0.01 (AlPcS_4_@FNPs loaded MSCs) at day 28 (**d**). Histological analysis of tumor tissues after treatments: H&E, *K*_i_-67 and TUNEL staining (scale bar = 100 μm, black arrow = necrotic areas) (**e**). For this study a total of 18 mice were used, mice were divided in 4 group as followed: mice treated with PBS (*n* = 3), with AlPcS_4_ alone (*n* = 3), with AlPcS_4_@FNPs NPs alone (*n* = 6) and with AlPcS_4_@FNPs loaded MSCs (*n* = 6)

The distribution and frequency of cells that survived the PDT treatment in the 1:7 ratio spheroids were further evaluated by toluidine blue staining on semi-thin slices taken across the entire spheroid at different levels (Figure 5S, Additional file 7). Interestingly, we observed that viable cells were predominantly located at the external rim or in the polar region of the spheroid, while internal areas showed necrosis or morphological alterations in the majority of cells. In the spheroids composed by unloaded MSCs and MG-63, no difference in term of viability was observed between irradiated or not irradiated samples (data not shown).

#### PDT efficacy in an ectopic murine osteosarcoma model

The effect of irradiation of AlPcS_4_@FNPs loaded MSCs was further investigated in an ectopic OS mouse model.

After 2 weeks from Saos-2-Luc/MSCs co-injection, subcutaneous tumors were visible. A volume of 50 μl of AlPcS_4_@FNPs alone or loaded in MSCs were administrated intra-tumorally, as well as PBS or AlPcS_4_ alone (control groups) (timeline in Fig. 5a). The localization of the nanoparticles (NPs) within the tumor was monitored by taking advantage of AlPcS_4_ fluorescence, using the IVIS Lumina II System. As shown in Fig. 5b the AlPcS_4_@FNP distribution was more localized around the site of injection when delivered by MSCs, than when NPs were injected alone. Twenty-four hours after the intra-tumor injection, the tumor was exposed to irradiation for 20 min. This cycle of treatment was repeated once a week for 2 weeks. As shown in Fig. 5c and d, after the first irradiation (day 21), no significant difference was observed among all groups. The second photoirradiation, instead, delayed the tumor growth in mice treated with AlPcS_4_@FNPs alone (− 65 ± 14%, *p* < 0.001) or with AlPcS_4_@FNPs loaded MSCs (− 62 ± 13%, *p* < 0.01) (day 28). As side effect of the PDT, a circumscribed dark eschar on the mouse skin was observed a few days after photoirradiation on the tumor surface only in mice injected with AlPcS_4_@FNPs alone but not in any of the other groups (Figure 6S, Additional file 8). One week after the last treatment, the reduction on the tumor growth was further enhanced for both AlPcS_4_@FNPs (− 72 ± 10%, *p* < 0.0001) and AlPcS_4_@FNPs loaded MSCs (− 68 ± 10%, *p* < 0.001).

Following the last BLI acquisition, mice were euthanized, and tumors were explanted and processed for histology. The anti-tumor effect of the PDT treatment in combination with AlPcS_4_@FNPs and AlPcS_4_@FNPs loaded MSCs injections were assessed with Hematoxylin and Eosin (H&E), *K*_i_-67 and TUNEL staining. H&E and TUNEL staining on tumor histological sections revealed areas of necrosis (highlighted in Fig. 5e by black arrow). In particular, we identified degraded areas composed predominantly of necrotic/apoptotic cells in tissue sections from AlPcS_4_@FNPs and AlPcS_4_@FNPs loaded MSCs photoirradiated samples. Instead, mice treated with PBS or free AlPcS_4_ had an extensive *K*_i_-67 staining, in fact almost all the cells showed uniform brown color, suggesting that the photoirradiation did not affect OS cell proliferation. However, cell proliferation decreased in both AlPcS_4_@FNPs and AlPcS_4_@FNPs loaded MSCs treated groups as shown in Fig. 5e, where the number of cells expressing *K*_i_-67 is greatly reduced.

In summary, we showed that under these experimental conditions, photostimulation treatments of intra-tumorally injected AlPcS_4_@FNP loaded in MSCs were able to kill OS cells and decrease tumor burden compared to both control groups (PBS and free AlPcS_4_). Moreover, the internalization of the AlPcS_4_@FNPs by the MSCs improved the local concentration of NPs at the injection site, eventually reducing side effects when compared to AlPcS_4_@FNP alone.

## Discussion

Despite the effort made by clinicians in the last 30 years, 30% of osteosarcoma (OS) patients still do not respond to standard treatments, succumbing to the disease [50]. Major issues for poor OS survival rate include the insurgence of distal metastases [51, 52], mainly localized in the lungs, and the development of multi drug resistance (MDR) [53, 54]. Possible strategies for improving OS patients’ survival rate include methods for selectively targeting the therapeutic agent to the tumor stroma, as well as the use of alternative therapeutic approaches able to either circumvent MDR or kill chemoresistant cells.

In this context, mesenchymal stromal cells (MSCs) are increasingly considered as an ideal vector for delivering antineoplastic drugs, because of their well-established ability to home towards the stroma of several primary and metastatic tumors [55, 56]. Indeed, MSCs have been used for the in vitro and in vivo delivery of, among others, diagnostic and therapeutic agents, small interfering RNA and nanoparticles [57]. In particular, several authors have shown that MSCs easily internalize different types of nanoparticles [27] and can reach the tumor, showing limited or no toxicity effect of NPs to MSCs [58, 59]. Additionally, other authors have investigated whether MSCs can transport nanoparticles for therapeutic purposes [22, 60, 61].

Among alternative cancer treatments, photodynamic therapy (PDT) has been successfully used to kill OS cells in vitro [35, 37, 62–65]; in particular Kusuzaki et al. has shown that PDT is also able to kill multidrug resistant OS cells [66]. In vivo the effectiveness of PDT has been demonstrated in OS animal models [34, 67, 68]. Moreover, PDT has been previously successfully used to treat sarcomas in a group of patients in which PDT inhibits the local recurrence after intralesional tumor resection [38, 69–71].

Our results successfully demonstrate that the internalization of AlPcS_4_@FNPs into MSCs takes place in 1 h, and that particles are retained in the cells for at least 3 days. This result is in line with Roger et al. work, where they demonstrated that PLA nanoparticles are internalized by MSCs up to 100% within 1 h, and that the particles are retained for at least 3 days [23, 24]. This aspect is particularly significant in view of clinical application since a 3 days interval is compatible with the migration of MSCs to the tumor from the site of the injection [72].

The awareness recently acquired that a monolayer culture is not predictive of in vivo results, prompted us to find an ex vivo model able to simulate in vivo physiology [73]. Tumor spheroids are an established model to investigate novel cancer treatments, as they provide a better recapitulation of tumor pathophysiological aspects, such as the in vivo-like differentiation pattern due to the appropriate 3D extracellular matrix (ECM) assembly and the complex cell–matrix and cell–cell interactions [74, 75]. Particularly, in our case the thickness of the cell aggregate (~ 400 μm of diameter), together with the presence of extracellular matrix and the inevitable oxygen gradient, provided a more challenging model for the PDT treatment. In this model, we clearly demonstrated that the efficacy of the photoactivation of AlPcS_4_@FNPs loaded MSCs depends on the ratio between MSCs and OS cells. As expected, this result suggested that the in vivo efficacy of this system will strongly depend on both the tumor’s dimensions and on the number of loaded MSCs that will reach the neoplastic region.

Additionally, by combining the results obtained from the APT content analysis, the Live&Dead staining assay and the TEM microscopy studies, we were able to establish that by decreasing the MSCs:OS ratio from 1:1 to 1:7, cell death is far higher in the center of the spheroid as respect to the outer region. This observation could be explained by the outgrowth of OS cells in the 4 days of spheroids formation that would ultimately confine MSCs in the more internal part of the spheroid. A similar distribution of MSCs in a spheroid model has been observed by Zhang et al. in melanoma cell spheroids [25].

As a starting point to test the efficacy in vivo of AlPcS_4_@FNPs loaded MSCs, an ectopic OS model was developed. An arbitrary dose of MSCs loaded with AlPcS_4_@FNPs was selected and injected intra-tumorally and the efficacy of two photoactivation cycles was tested. Compared to the control groups (PBS and AlPcS_4_ alone), after the second photoirradiation, tumor growth was reduced in both groups (AlPcS_4_@FNPs and AlPcS_4_@FNPs@MSCs, 72 and 68% respectively). Results observed by luminescence analysis were confirmed by histological changes in the treated tumor sections. Clear evidence of apoptosis, observed by H&E and TUNEL staining, were associated with PDT treatment in combination with AlPcS_4_@FNPs injection (alone or loaded in MSCs), supporting the role of our system in the cell-killing process after photoirradiation.

The results described herein demonstrate that the AlPcS_4_@FNPs@MSCs system is very promising for treating OS tumors. However, treatment outcome could be improved by increasing PDT efficacy, by either performing more irradiation cycles or/and by optimizing the AlPcS_4_@FNPs loaded MSCs dose as well the irradiation conditions. Importantly, even if a reduction of tumor growth was demonstrated in both groups of mice, i.e. AlPcS_4_@FNPs alone and AlPcS_4_@FNPs@MSCs, in the mice injected with AlPcS_4_@FNPs alone, the NPs had a larger distribution as shown in Fig. 5a, thus supporting the tumor targeting effect of MSCs. Furthermore, it is interesting to notice that only mice injected with AlPcS_4_@FNPs alone, displayed skin superficial burns, probably generated by an excessive local heating due to an over-concentration of the particles in the skin and their subsequent photoactivation, as already observed by others [76]. This data further supports the potential advantage of using MSCs as a delivery system in terms of selective localization at the target tissue, which in turn allows the control of unwanted side effects.

## Conclusions

In summary, our results obtained both in vitro and in vivo show that the use of MSCs to deliver photoactivatable NPs together with PDT can be a promising treatment for OS. However, our findings indicate that MSCs loaded with AlPcS_4_@FNPs could be especially promising when used either in patients that have developed chemoresistance or when the tumor is small and located in inoperable sites. To conclude, we believe that MSCs based PDT technology will impact the design of clinical trial for personalized treatment.

## Supplementary information

**Additional file 1: Supplementary Methods.** Human mesenchymal stromal cells (MSCs) characterization and potency assays.

**Additional file 2: Table 1S.** MSCs characterization and in-process data. The table shows data related to isolation (CFU assay; cell yield); expansion (CPDs); osteogenic, adipogenic, chondrogenic differentiation assays; and immunophenotype profiles of the 5 isolated MSCs lines.

**Additional file 3: Table 2S.** FNPs hydrodynamic diameters. Hydrodynamic FNPs diameter measurements by dynamic light scattering (mQ water). Hydrodynamic diameter of the nanoparticles was determined by photon correlation spectroscopy (PCS) at 25 °C using a NanoBrook Omni Particle Size Analyzer (Brookhaven Instruments Corporation, USA) equipped with a 35 mW red diode laser (nominal 640 nm wave- length). **Table 3S.** Zeta-potential measurements of FNPs (mQ water). Zeta-potential was measured at 25 °C using a NanoBrook Omni Particle Size Analyzer (Brookhaven Instruments Corporation, USA) equipped with a 35 mW red diode laser (nominal 640 nm wave- length). **Figure 1S.** FNPs scanning electron microscope (SEM) image. Particles morphology was studied using the EVO LS 10 LaB6 scanning electron microscopy (SEM) (Zeiss, Italy) with an acceleration voltage of 5 kV and a working distance of 5 mm. The samples were sputter coated under vacuum with a thin layer (10–30 Å) of gold. Z-range = 63 ± 13 nm.

**Additional file 4: Figure 2S.** 3D model characterization. Diameter measures (mean ± SD, *n* = 6) from day1 to day15 of spheroids prepared with the 3 OS cells lines (a) and representative images of spheroids prepared with 10^4^ OS cells (b). **Table 2S.** Measures of 3D model morphological properties. Diameter and roughness coefficient measures from brightfield images of spheroids obtained from different OS cell lines after 3 days of culture in low attachment 96-well plate.

**Additional file 5: Figure 3S.** FNPs retention inside multicellular spheroids. Representative images of spheroids composed by MG-63 and AlPcS_4_@FNPs loaded MSC in 1:1 ratio, after 3 and 8 days of culture, showing the green fluorescent emission of AlPcS_4_@FNPs superimposed on the brightfield images of the whole spheroids (a). Quantification of fluorescence intensity inside the spheroids (*n* = 8) at the indicated timepoints; the spheroids’ perimeters traced on brightfield images were superimposed to corresponding fluorescent images to select a ROI and obtain the total intensity of green channel in the selected area (b).

**Additional file 6: Figure 4S.** Survival rate tested in 5 MSC lines isolated from patients. Quantification of vitality by ATP assay (Cell Titer Glo 3D) 24 h after 10 min PDT in 5 MSCs cell lines loaded with 90μg/ml AlPcS_4_@NPs in co-culture with MG-63 cells in ratio 1:1.

**Additional file 7: Figure 5S.** Full depth analysis of resin embedded spheroids. Toluidine blue stained semi-thin slices of one multicellular spheroid composed by AlPcS_4_@NPs loaded MSCs and MG-63 in a 1:7 ratio after 10 min irradiation (scale bar = 100 μm). Asterisks mark the areas where intact cells were observed. Slices correspond to different axial planes during cutting session as schematically explained in the first row. The location of each slice on the vertical axis depicted in the cartoon is approximated and arbitrary, since the orientation of the spheroids during cutting cannot be correlated with the orientation of the spheroid during PDT treatment. A central slice of a not irradiated spheroid is showed as positive control for the staining (blue frame).

**Additional file 8: Figure 6S.** Representative imaging of photodynamic therapy effect on tumor bearing mice. Representative images of photoirradiated tumor on mouse left flank. Characteristic burn/scab formation was observed 24 h after second treatment in mice injected with AlPcS_4_@FNPs alone (white arrowhead).

## Data Availability

The datasets used and/or analyzed during the current study are available from the corresponding author on reasonable request.

## Abbreviations

AlPcS_4_: Tetra-sulfonated aluminum phthalocyanine
AlPcS_4_@FNPs: FNPs decorated with AlPcS_4_
AlPcS_4_@FNPs@MSCs: AlPcS_4_@FNPs loaded MSCs
AlPcS_4_@NPs: NPs decorated with AlPcS_4_
BLI: Bioluminescence imaging
FNPs: Poly-methyl methacrylate core-shell fluorescent nanoparticles
MSCs: Human mesenchymal stromal cells
NPs: Poly-methyl methacrylate core-shell nanoparticles
OS: Osteosarcoma
PDT: Photodynamic therapy
PS: Photosensitizer
TEM: Transmission electron microscopy

## References

[1] Bacci G, Bertoni F, Longhi A, Ferrari S, Forni C, Biagini R (2003). Neoadjuvant chemotherapy for high-grade central osteosarcoma of the extremity. Cancer.

[2] Longhi A, Errani C, De Paolis M, Mercuri M, Bacci G (2006). Primary bone osteosarcoma in the pediatric age: state of the art. Cancer Treat Rev.

[3] Li W, Zhang S (2008). Survival of patients with primary osteosarcoma and lung metastases. J BUON.

[4] Anderson ME (2016). Update on survival in osteosarcoma. Orthop Clin North Am.

[5] Anninga JK, Gelderblom H, Fiocco M, Kroep JR, Taminiau AHM, Hogendoorn PCW (2011). Chemotherapeutic adjuvant treatment for osteosarcoma: where do we stand?. Eur J Cancer.

[6] Zhang Y, Yang J, Zhao N, Wang C, Kamar S, Zhou Y (2018). Progress in the chemotherapeutic treatment of osteosarcoma (review). Oncol Lett.

[7] Rajkumar T, Yamuna M (2008). Multiple pathways are involved in drug resistance to doxorubicin in an osteosarcoma cell line. Anti-Cancer Drugs.

[8] Squillaro T, Peluso G, Galderisi U (2016). Clinical trials with mesenchymal stem cells: an update. Cell Transplant.

[9] Kidd S, Spaeth E, Dembinski JL, Dietrich M, Watson K, Klopp A (2009). Direct evidence of mesenchymal stem cell tropism for tumor and wounding microenvironments using in vivo bioluminescent imaging. Stem Cells.

[10] Studeny M, Marini FC, Champlin RE, Zompetta C, Fidler IJ, Andreeff M (2002). Bone marrow-derived mesenchymal stem cells as vehicles for interferon-β delivery into tumors. Cancer Res.

[11] D’souza N, Rossignoli F, Golinelli G, Grisendi G, Spano C, Candini O (2015). Mesenchymal stem/stromal cells as a delivery platform in cell and gene therapies. BMC Med.

[12] Johnson PWM (2015). Masses in the mediastinum: primary mediastinal lymphoma and intermediate types. Hematol Oncol.

[13] Komarova S, Kawakami Y, Stoff-Khalili MA, Curiel DT, Pereboeva L (2006). Mesenchymal progenitor cells as cellular vehicles for delivery of oncolytic adenoviruses. Mol Cancer Ther.

[14] Moreno R, Rojas LA, Villellas FV, Soriano VC, García-Castro J, Fajardo CA (2017). Human menstrual blood-derived mesenchymal stem cells as potential cell carriers for oncolytic adenovirus. Stem Cells Int.

[15] Pessina A, Bonomi A, Coccè V, Invernici G, Navone S, Cavicchini L (2011). Mesenchymal stromal cells primed with paclitaxel provide a new approach for cancer therapy. PLoS One.

[16] Petrella F, Rimoldi I, Rizzo S, Spaggiari L (2017). Mesenchymal stromal cells for antineoplastic drug loading and delivery. Medicine (Basel, Switzerland).

[17] Sonabend AM, Ulasov IV, Tyler MA, Rivera AA, Mathis JM, Lesniak MS (2008). Mesenchymal stem cells effectively deliver an oncolytic adenovirus to intracranial glioma. Stem Cells.

[18] Studeny M, Marini FC, Dembinski JL, Zompetta C, Cabreira-Hansen M, Bekele BN (2004). Mesenchymal stem cells: potential precursors for tumor stroma and targeted-delivery vehicles for anticancer agents. J Natl Cancer Inst.

[19] Pessina A, Leonetti C, Artuso S, Benetti A, Dessy E, Pascucci L (2015). Drug-releasing mesenchymal cells strongly suppress B16 lung metastasis in a syngeneic murine model. J Exp Clin Cancer Res.

[20] Scioli MG, Artuso S, D’Angelo C, Porru M, D’Amico F, Bielli A (2018). Adipose-derived stem cell-mediated paclitaxel delivery inhibits breast cancer growth. PLoS One.

[21] Gao Z, Zhang L, Hu J, Sun Y (2013). Mesenchymal stem cells: a potential targeted-delivery vehicle for anti-cancer drug, loaded nanoparticles. Nanomedicine.

[22] Layek B, Sadhukha T, Panyam J, Prabha S (2018). Nano-engineered mesenchymal stem cells increase therapeutic efficacy of anticancer drug through true active tumor targeting. Mol Cancer Ther.

[23] Paris JL, de la Torre P, Manzano M, Cabañas MV, Flores AI, Vallet-Regí M (2016). Decidua-derived mesenchymal stem cells as carriers of mesoporous silica nanoparticles. In vitro and in vivo evaluation on mammary tumors. Acta Biomater.

[24] Roger M, Clavreul A, Venier-Julienne M-C, Passirani C, Sindji L, Schiller P (2010). Mesenchymal stem cells as cellular vehicles for delivery of nanoparticles to brain tumors. Biomaterials.

[25] Zhang T-Y, Huang B, Wu H-B, Wu J-H, Li L-M, Li Y-X (2015). Synergistic effects of co-administration of suicide gene expressing mesenchymal stem cells and prodrug-encapsulated liposome on aggressive lung melanoma metastases in mice. J Control Release.

[26] Duchi S, Sotgiu G, Lucarelli E, Ballestri M, Dozza B, Santi S (2013). Mesenchymal stem cells as delivery vehicle of porphyrin loaded nanoparticles: effective photoinduced in vitro killing of osteosarcoma. J Control Release.

[27] Zhao Y, Tang S, Guo J, Alahdal M, Cao S, Yang Z (2017). Targeted delivery of doxorubicin by nano-loaded mesenchymal stem cells for lung melanoma metastases therapy. Sci Rep.

[28] Saulite L, Dapkute D, Pleiko K, Popena I, Steponkiene S, Rotomskis R (2017). Nano-engineered skin mesenchymal stem cells: potential vehicles for tumour-targeted quantum-dot delivery. Beilstein J Nanotechnol.

[29] Zhang J, Jiang C, Figueiró Longo JP, Azevedo RB, Zhang H, Muehlmann LA (2018). An updated overview on the development of new photosensitizers for anticancer photodynamic therapy. Acta Pharm Sin B.

[30] Lucky SS, Soo KC, Zhang Y (2015). Nanoparticles in photodynamic therapy. Chem Rev.

[31] Kwiatkowski S, Knap B, Przystupski D, Saczko J, Kędzierska E, Knap-Czop K (2018). Photodynamic therapy – mechanisms, photosensitizers and combinations. Biomed Pharmacother.

[32] van Straten D, Mashayekhi V, de Bruijn H, Oliveira S, Robinson D (2017). Oncologic photodynamic therapy: basic principles, current clinical status and future directions. Cancers (Basel).

[33] de Miguel GC, Abrantes AM, Laranjo M, Grizotto AYK, Camporeze B, Pereira JA (2018). A new therapeutic proposal for inoperable osteosarcoma: photodynamic therapy. Photodiagn Photodyn Ther.

[34] Meier D, Botter SM, Campanile C, Robl B, Gräfe S, Pellegrini G (2017). Foscan and foslip based photodynamic therapy in osteosarcoma in vitro and in intratibial mouse models. Int J Cancer.

[35] Reidy K, Campanile C, Muff R, Born W, Fuchs B (2012). mTHPC-mediated photodynamic therapy is effective in the metastatic human 143B osteosarcoma cells. Photochem Photobiol.

[36] Satonaka H, Kusuzaki K, Akeda K, Tsujii M, Iino T, Uemura T (2011). Acridine orange inhibits pulmonary metastasis of mouse osteosarcoma. Anticancer Res.

[37] White B, Rossi V, Baugher PJ (2016). Aminolevulinic acid-mediated photodynamic therapy causes cell death in MG-63 human osteosarcoma cells. Photomed Laser Surg.

[38] Yu W, Zhu J, Wang Y, Wang J, Fang W, Xia K (2017). A review and outlook in the treatment of osteosarcoma and other deep tumors with photodynamic therapy: from basic to deep. Oncotarget.

[39] Matsubara T, Kusuzaki K, Matsumine A, Satonaka H, Shintani K, Nakamura T (2008). Methylene blue in place of acridine orange as a photosensitizer in photodynamic therapy of osteosarcoma. In Vivo.

[40] Satonaka H, Kusuzaki K, Matsubara T, Shintani K, Wakabayashi T, Nakamura T (2007). Flash wave light strongly enhanced the cytocidal effect of photodynamic therapy with acridine orange on a mouse osteosarcoma cell line. Anticancer Res.

[41] Duchi S, Ramos-Romero S, Dozza B, Guerra-Rebollo M, Cattini L, Ballestri M (2016). Development of near-infrared photoactivable phthalocyanine-loaded nanoparticles to kill tumor cells: an improved tool for photodynamic therapy of solid cancers. Nanomed Nanotechnol, Biol Med.

[42] Kusuzaki K, Murata H, Matsubara T, Satonaka H, Wakabayashi T, Matsumine A (2007). Review. Acridine orange could be an innovative anticancer agent under photon energy. In Vivo.

[43] Agostinis P, Berg K, Cengel KA, Foster TH, Girotti AW, Gollnick SO (2011). Photodynamic therapy of cancer: an update. CA Cancer J Clin.

[44] Monasterolo C, Ballestri M, Sotgiu G, Guerrini A, Dambruoso P, Sparnacci K (2012). Sulfonates-PMMA nanoparticles conjugates: a versatile system for multimodal application. Bioorganic Med Chem.

[45] Pierini M, Di Bella C, Dozza B, Frisoni T, Martella E, Bellotti C (2013). The posterior iliac crest outperforms the anterior iliac crest when obtaining mesenchymal stem cells from bone marrow. J Bone Joint Surg Am.

[46] Bara JJ, Richards RG, Alini M, Stoddart MJ (2014). Concise review: bone marrow-derived mesenchymal stem cells change phenotype following in vitro culture: implications for basic research and the clinic. Stem Cells.

[47] Whitfield MJ, Lee WCJ, Van Vliet KJ (2013). Onset of heterogeneity in culture-expanded bone marrow stromal cells. Stem Cell Res.

[48] Reger RL, Prockop DJ (2014). Should publications on mesenchymal stem/progenitor cells include in-process data on the preparation of the cells?. Stem Cells Transl Med.

[49] Phinney DG, Kopen G, Righter W, Webster S, Tremain N, Prockop DJ (1999). Donor variation in the growth properties and osteogenic potential of human marrow stromal cells. J Cell Biochem.

[50] Miller BJ, Cram P, Lynch CF, Buckwalter JA (2013). Risk factors for metastatic disease at presentation with osteosarcoma. J Bone Jt Surg Am.

[51] Rasalkar DD, Chu WCW, Lee V, Paunipagar BK, Cheng FWT, Li CK (2011). Pulmonary metastases in children with osteosarcoma: characteristics and impact on patient survival. Pediatr Radiol.

[52] Matsubara E, Mori T, Koga T, Shibata H, Ikeda K, Shiraishi K (2015). Metastasectomy of pulmonary metastases from osteosarcoma: prognostic factors and indication for repeat metastasectomy. J Respir Med.

[53] Yang X, Yang P, Shen J, Osaka E, Choy E, Cote G (2014). Prevention of multidrug resistance (MDR) in osteosarcoma by NSC23925. Br J Cancer.

[54] Chou AJ, Gorlick R (2006). Chemotherapy resistance in osteosarcoma: current challenges and future directions. Expert Rev Anticancer Ther.

[55] Kalimuthu S, Zhu L, Oh JM, Gangadaran P, Lee HW, Baek SH (2018). Migration of mesenchymal stem cells to tumor xenograft models and in vitro drug delivery by doxorubicin. Int J Med Sci.

[56] Xie C, Yang Z, Suo Y, Chen Q, Wei D, Weng X (2017). Systemically infused mesenchymal stem cells show different homing profiles in healthy and tumor mouse models. Stem Cells Transl Med.

[57] Chulpanova DS, Kitaeva KV, Tazetdinova LG, James V, Rizvanov AA, Solovyeva VV (2018). Application of mesenchymal stem cells for therapeutic agent delivery in anti-tumor treatment. Front Pharmacol.

[58] Loebinger MR, Kyrtatos PG, Turmaine M, Price AN, Pankhurst Q, Lythgoe MF (2009). Magnetic resonance imaging of mesenchymal stem cells homing to pulmonary metastases using biocompatible magnetic nanoparticles. Cancer Res.

[59] Liu X, Yang Z, Sun J, Ma T, Hua F, Shen Z (2019). A brief review of cytotoxicity of nanoparticles on mesenchymal stem cells in regenerative medicine. Int J Nanomedicine.

[60] Huang L, Xu C, Xu P, Qin Y, Chen M, Feng Q (2019). Intelligent photosensitive mesenchymal stem cells and cell-derived microvesicles for photothermal therapy of prostate cancer. Nanotheranostics.

[61] Chung T-HH, Hsu S-CC WS-HH, Hsiao J-KK, Lin C-PP, Yao M (2018). Dextran-coated iron oxide nanoparticle-improved therapeutic effects of human mesenchymal stem cells in a mouse model of Parkinson’s disease. Nanoscale.

[62] Coupienne I, Fettweis G, Piette J (2011). RIP3 expression induces a death profile change in U2OS osteosarcoma cells after 5-ALA-PDT. Lasers Surg Med.

[63] Nagai Y, Aizawa S, Iriuchishima T, Goto B, Nagaoka M, Tokuhashi Y (2014). Phototoxic effect of na-pheophorbide a toward osteosarcoma cells in vitro using a laser diode. Photomed Laser Surg.

[64] Tsai S-R, Yin R, Huang Y-Y, Sheu B-C, Lee S-C, Hamblin MR (2015). Low-level light therapy potentiates NPe6-mediated photodynamic therapy in a human osteosarcoma cell line via increased ATP. Photodiagn Photodyn Ther.

[65] Tu P, Huang Q, Ou Y, Du X, Li K, Tao Y (2016). Aloe-emodin-mediated photodynamic therapy induces autophagy and apoptosis in human osteosarcoma cell line MG-63 through the ROS/JNK signaling pathway. Oncol Rep.

[66] Kusuzaki K, Minami G, Takeshita H, Murata H, Hashiguchi S, Nozaki T (2000). Photodynamic inactivation with acridine orange on a multidrug-resistant mouse osteosarcoma cell line. Jpn J Cancer Res.

[67] Zeng H, Sun M, Zhou C, Yin F, Wang Z, Hua Y (2013). Hematoporphyrin monomethyl ether-mediated photodynamic therapy selectively kills sarcomas by inducing apoptosis. PLoS One.

[68] Nomura J, Yanase S, Matsumura Y, Nagai K, Tagawa T (2004). Efficacy of combined photodynamic and hyperthermic therapy with a new light source in an in vivo osteosarcoma tumor model. J Clin Laser Med Surg.

[69] Nakamura T, Kusuzaki K, Matsubara T, Murata H, Hagi T, Asanuma K (2018). Long-term clinical outcome in patients with high-grade soft tissue sarcoma who were treated with surgical adjuvant therapy using acridine orange after intra-lesional or marginal resection. Photodiagn Photodyn Ther.

[70] Carina V, Costa V, Sartori M, Bellavia D, De Luca A, Raimondi L (2019). Adjuvant biophysical therapies in osteosarcoma. Cancers (Basel)..

[71] Sun M, Zhou C, Zeng H, Puebla-Osorio N, Damiani E, Chen J (2015). Hiporfin-mediated photodynamic therapy in preclinical treatment of osteosarcoma. Photochem Photobiol.

[72] Vegh I, Grau M, Gracia M, Grande J, de la Torre P, Flores AI (2013). Decidua mesenchymal stem cells migrated toward mammary tumors in vitro and in vivo affecting tumor growth and tumor development. Cancer Gene Ther.

[73] Kucinska M, Murias M, Nowak-Sliwinska P (2017). Beyond mouse cancer models: three-dimensional human-relevant in vitro and non-mammalian in vivo models for photodynamic therapy. Mutat Res.

[74] Friedrich J, Seidel C, Ebner R, Kunz-Schughart LA (2009). Spheroid-based drug screen: considerations and practical approach. Nat Protoc.

[75] Huang B-W, Gao J-Q (2018). Application of 3D cultured multicellular spheroid tumor models in tumor-targeted drug delivery system research. J Control Release.

[76] Silva ZS, Bussadori SK, Fernandes KPS, Huang Y-Y, Hamblin MR. Animal models for photodynamic therapy (PDT). Biosci Rep. 2015;35(6). 10.1042/BSR20150188. 10.1042/BSR20150188 PMC4643327 26415497

