# Supplementary material for: Mesenchymal stromal cells mediated delivery of photoactive nanoparticles inhibits osteosarcoma growth in vitro and in a murine in vivo ectopic model
Source: J Exp Clin Cancer Res. 2020 Feb 22;39:40. doi: 10.1186/s13046-020-01548-4 (PMC7036176; doi:10.1186/s13046-020-01548-4)
Supplement: Supplementary file 1 — Additional file 1: Supplementary Methods. Human mesenchymal stromal cells (MSCs) characterization and potency assays. [file 13046_2020_1548_MOESM1_ESM.pdf]

Supplementary Methods

Human mesenchymal ~~stem~~ stromal cells (MSCs) characterization and potency assays.

Fibroblasts-Colony forming unit (CFU-F) assay.

This assay was used to assess the clonogenicity potential of bone marrow samples before and after the gradient separation process. 1.5 x 10^5 mononucleated cells were plated in 100mm culture dish with 15mL of α-MEM, supplemented with FBS 20% and GlutaMAX 1%, and maintained for 2 weeks at 37°C in 5% CO_2 without media change. The colonies of BM MSCs precursors were fixed with methanol at room temperature for 10 min and stained with methylene blue 1% in borate buffer 0.01 M (pH 8.5). Individual colonies derived from a single precursor were counted independently by two investigators. All experiments were performed in duplicate.

Immunophenotype.

MSCs at passage 3 were tested for the expression of specific immunophenotypic markers: CD14, CD31 (pecam-1), CD34, CD45, CD73 and CD90 (thy-1). Briefly, 1x10^5 cells were dispensed in a 15mL tube and resuspended in FACS buffer (5% FBS in D-PBS). 5µL of the specific FITC-conjugate antibody or a mouse IgG1 k isotype control (Affimetrix, Santa Clara, California, United States) were added to the cell suspension, mixed gently and incubated for 30min at 4°C in the dark. Then, cells were centrifuged at 1500rpm for 3 min, washed once with FACS buffer, resuspended in 200µL of FACS buffer and finally analyzed using FACScalibur device.

Osteogenic differentiation.

At passage 3, MSCs were seeded on a six well plate at a density of 5.2x10^4 cells/cm^2 in α-MEM supplemented with 2%FBS. Cells were let to adhere overnight, then a differentiating mix composed of β-glicerophosphate, dexamethasone and ascorbic acid (all from Sigma Aldrich) was added to the medium to a final concentration of 10mM, 100nM and 284µM, respectively. Medium with or without the differentiating mix was change twice a week. After 14 days of treatment, cells were fixed in 70% ethanol for 1 hour, washed twice with D-PBS and stained with Alizarin Red S 40 mM solution (pH 4-4,2) for 15 min at RT to reveal the mineralized matrix. After removing the Alizarin Red excess with multiple rinsing with distilled water, images were acquired using an inverted microscope (Eclipse TE 2000, Nikon). The Alizarin Red bound to the matrix was then solubilized on an orbital shaker for 15 min, adding 1 ml of 10% acetyl-pyridinium (Sigma Aldrich) in sodium phosphate buffer (10 mM pH 7,0). Optical density at 562nm was measured using a multiplate reader (Synergy HT, BioTek,Winooski, VT, USA) using OD_{562nm} of 10% acetyl-pyridinium for background correction.

Adipogenic differentiation.

MSCs adipogenic potential was assessed as previously described by Barbero *et al.*(1). Briefly, cells were seeded at a density of 3x10^3 cells/cm^2 in a 24-well plate and cultured in DMEM-HG with 10%FBS until confluence. The basal medium was then supplemented with 10µg/mL insulin, 1µM dexamethasone, 200µM indomethacin and 500µM 3-isobutyl-1-methyl xanthine (all from Sigma Aldrich) for 72 hours and subsequently with 10µg/mL insulin for 24 hours. This 96-hours cycle of treatments was repeated five times. Control cells where cultured for the same period in basal medium. At the end, MSC cultures were washed with PBS, fixed in 10% neutral buffered formalin for 10 min and stained with a mixture of three volumes of

0.3% Oil Red O solution in isopropanol and two volumes of ddH_2O for 15 min at room temperature on an orbital shaker. After several washes in PBS and water, microphotographs were taken; then Oil Red O was solubilized with 100% isopropanol and the optical density at 500 nm was measured using a multiplate reader using OD of isopropanol for background correction (Synergy HT, BioTek,Winooski, VT, USA) (2)

Chondrogenic differentiation.

To assess chondrogenic potential cells were culture as micromasses (pellet culture). More specifically, $2.5x10^5$ MSCs were dispensed in a 15mL tube in 500µL of a commercial chondrogenic induction medium (StemPro Chondrogenesis Differentiation Kit, Life Technologies). Cells were centrifuged for 5 min at 460g and leave at 37°C 5%CO_2 to induce pellet formation. The pellet culture was maintained in control (DMEM-HG 10%FBS) or chondrogenic medium for 21 days, changing the medium twice a week. At the end of treatment, pellets were digested with papain solution (0.4 mg/mL papain in 200 mM pH 6.5 sodium phosphate buffer, containing 2 mM N-acetyl-L-cysteine and 2 mM Na_2EDTA) at 65°C for 3 hours, then centrifuged at 10,000 g for 10 min. Supernatants were collected and the glycosamminoglycan content was evaluated using the Blyscan Sulfated Glycosaminoglycan Assay kit (Biocolor Ltd, Carrickfergus, United Kindom) following the manufacturer's instructions (3,4).

MSCs proliferation

Cell number and viability were assessed at each passage using a NucleoCounter® device (ChemoMetec, Allerød, Denmark) that detects non-viable cells by propidium iodide nuclear staining and determines cell viability by calculating the ratio of non-viable to total cell numbers. The number of population doublings (PDs) for each passage was calculated using the formula: $log_2(N_1/N_0)$, where N_0 is the number of cells seeded and N_1 the number of cells harvested when cells cover about the 70% of culture vessel surface. Cumulative population doubling (CPD) refers to the sum of PDs over the passages.

Spheroids analysis

Brightfield and epifluorescence images were acquired with optical microscope Nikon Eclipse TE2000-U. The inbuilt Nikon NIS-D software was used to trace the spheroid perimeter (with automated algorithm) and to calculate diameters and roughness coefficients. For fluorescence analysis of FNPs retention, the traced perimeters were used as ROI to calculate the Sum Intensity of the area in the Green channel.

References

1. Barbero A, Ploegert S, Heberer M, Martin I. Plasticity of clonal populations of dedifferentiated adult human articular chondrocytes. Arthritis Rheum. 2003 May;48(5):1315–25.

2. Donzelli E, Lucchini C, Ballarini E, Scuteri A, Carini F, Tredici G, et al. ERK1 and ERK2 are involved in recruitment and maturation of human mesenchymal stem cells induced to adipogenic differentiation. J Mol Cell Biol. 2011 Apr;3(2):123–31.

3. Lee J-S, Im G-I. Influence of chondrocytes on the chondrogenic differentiation of adipose stem cells. Tissue Eng Part A. 2010 Dec;16(12):3569–77.

4. Yoneno K, Ohno S, Tanimoto K, Honda K, Tanaka N, Doi T, et al. Multidifferentiation potential of mesenchymal stem cells in three-dimensional collagen gel cultures. J Biomed Mater Res - Part A. 2005;75(3):733–41.
