# Supplementary material for: Mesenchymal stromal cells mediated delivery of photoactive nanoparticles inhibits osteosarcoma growth in vitro and in a murine in vivo ectopic model
Source: J Exp Clin Cancer Res. 2020 Feb 22;39:40. doi: 10.1186/s13046-020-01548-4 (PMC7036176; doi:10.1186/s13046-020-01548-4)
Supplement: Supplementary file 2 — Additional file 2: Table 1S. MSCs characterization and in-process data. The table shows data related to isolation (CFU assay; cell yield); expansion (CPDs); osteogenic, adipogenic, chondrogenic differentiation assays; and immunophenotype profiles of the 5 isolated MSCs lines. [file 13046_2020_1548_MOESM2_ESM.pdf]

Table 1S. MSCs characterization and in-process data. –

The table shows data related to isolation (CFU assay; cell yield); expansion (CPDs); osteogenic, adipogenic, chondrogenic differentiation assays; and immunophenotype profiles of the 5 isolated MSCs lines.

MSCs ID# in the manuscript	Donor[a] (ID#/gender/age)	CFU-F/10^6 cells[b] (before/after isolation)	P0 yield[c] ($x10^6$)/days	P0→P1 yield[d] (harvested cells per cm^2/days)	P1→P2 yield[d] (harvested cells per cm^2/days)	P1 cumulative PDs[e]	P2 cumulative PDs[e]	P6 cumulative PDs[e]	P10 cumulative PDs[e]	P3 diff. score[f] (osteo/adipo/condro)
**1**	268/F/13	53/67	2.95/13	$1.39x10^4$/19	$1.42x10^4$/25	J+2.79	J+6.88	J+18.92	29.78	1/2/4
**2**	269/M/36	130/93	4.88/13	$2.55x10^4$/19	$2.53x10^4$/25	J+3.67	J+5.62	J+20.87	29.50	2/2/4
**3**	272/F/22	90/87	4.99/14	$2.35x10^4$/21	$2.18x10^4$/28	J+3.56	J+7.00	J+19.12	28.01	2/3/2
**4**	273/M/42	83/70	3.92/13	$1.70x10^4$/20	$9.62x10^3$/27	J+3.08	J+5.35	J+16.73	20.47	3/2/3
**5**	274/M/15	70/147	3.79/12	$2.61x10^4$/19	$1.73x10^4$/26	J+3.71	J+6.82	J+16.2	26.67	2/1/4

MSC ID#	P3 epitopes[g] (-)				P3 epitopes[g] (+)	
	**CD14**	**CD31**	**CD34**	**CD45**	**CD90**	**CD73**
**1**	2	2	6	14	100	95
**2**	1	1	3	9	100	93
**3**	1	1	2	5	100	88
**4**	0	0	12	5	100	98
**5**	0	0	0	0	100	97

[a]Donors identified by anonymous number, gender and age.
[b] CFU-f obtained as previously described from bone marrow sample (before isolation) and from mononuclear cells isolated by density gradient (after isolation)
[c]P0 yield defined as number of cells (as millions) obtained by plating mononuclear cells from Ficoll gradient at high density, (400000 cells per cm^2)/days cells were incubated.
[d]P1/P2 yields of defined by number of cells harvested per cm^2/days of incubation.
[e]Cumulative PDs defined as J+ observed value because number of population doublings required to generate P0 cells cannot be estimated.
[f] Scores of differentiation assays. Scores from 0 to 5 were defined comparing the results to the percentile originated from the lab's historical database (0 if <5th, 1 if between 5thand 25th, 2 if between 25th and 50th, 3 if between 50th and 75th, 3 if between 75th and 95th).
[g]Positive epitopes (CD14, CD31, CD34, CD45) and negative epitopes (CD73, CD90) cell percentages in passage 3 populations.
Abbreviations: CFU, colony-forming unit; F, female; M, male; PDs, population doublings
