# Supplementary material for: Mesenchymal stromal cells mediated delivery of photoactive nanoparticles inhibits osteosarcoma growth in vitro and in a murine in vivo ectopic model
Source: J Exp Clin Cancer Res. 2020 Feb 22;39:40. doi: 10.1186/s13046-020-01548-4 (PMC7036176; doi:10.1186/s13046-020-01548-4)
Supplement: Supplementary file 3 — Additional file 3: Table 2S. FNPs hydrodynamic diameters. Hydrodynamic FNPs diameter measurements by dynamic light scattering (mQ water). Hydrodynamic diameter of the nanoparticles was determined by photon correlation spectroscopy (PCS) at 25 °C using a NanoBrook Omni Particle Size Analyzer (Brookhaven Instruments Corporation, USA) equipped with a 35 mW red diode laser (nominal 640 nm wave- length). Table 3S. Zeta-potential measurements of FNPs (mQ water). Zeta-potential was measured at 25 °C using a NanoBrook Omni Particle Size Analyzer (Brookhaven Instruments Corporation, USA) equipped with a 35 mW red diode laser (nominal 640 nm wave- length). Figure 1S. FNPs scanning electron microscope (SEM) image. Particles morphology was studied using the EVO LS 10 LaB6 scanning electron microscopy (SEM) (Zeiss, Italy) with an acceleration voltage of 5 kV and a working distance of 5 mm. The samples were sputter coated under vacuum with a thin layer (10–30 Å) of gold. Z-range = 63 ± 13 nm. [file 13046_2020_1548_MOESM3_ESM.pdf]

Table 2S. **FNPs hydrodynamic diameters.**
Hydrodynamic FNPs diameter measurements by dynamic light scattering (mQ water). Hydrodynamic diameter of the nanoparticles was determined by photon ~~corre- lation~~ spectroscopy (PCS) at 25 °C using a NanoBrook Omni Particle Size Analyzer (Brookhaven Instruments Corporation, USA) equipped with a 35 mW red diode laser (nominal 640 nm wave- length).

Run	Eff. Diam. (nm)	Polydispersity
1	76.87	0.183
2	76.76	0.134
3	75.92	0.155
4	74.53	0.187
5	71.95	0.167
**Mean**:	75.21	0.165
**Std Err:**	0.92	0.010
**Std Dev**:	2.05	0.022

Table 3S. Zeta-potential measurements of FNPs (mQ water). Zeta-potential was measured at 25 °C using a NanoBrook Omni Particle Size Analyzer (Brookhaven Instruments Corporation, USA) equipped with a 35 mW red diode laser (nominal 640 nm wave- length).

Run	Zeta-potential (mV)
1	55.99
2	57.10
3	49.09
4	49.65
5	58.99
**Mean:**	54.16
**Std Err**:	1.80
**Std Dev:**	4.03

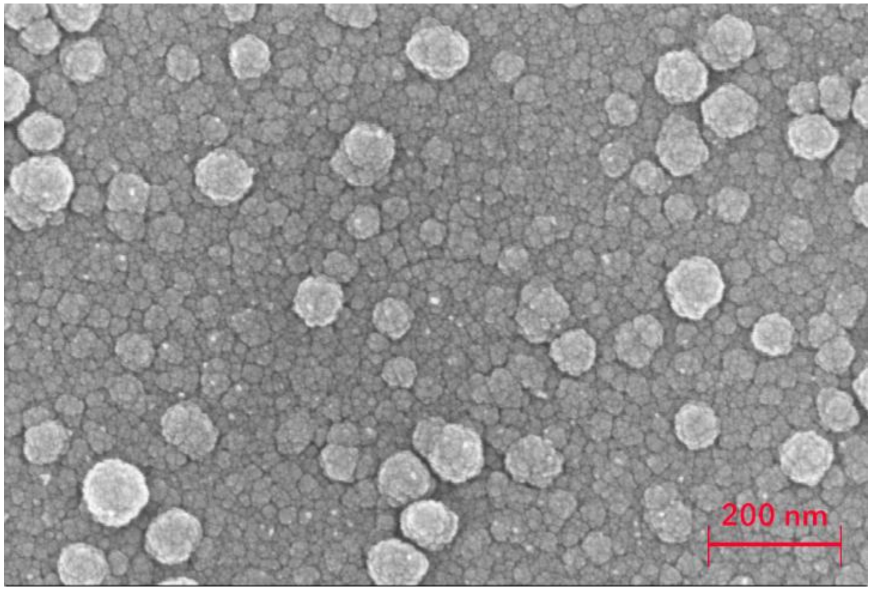

Figure 1S. **FNPs scanning electron microscope (SEM) image.**
Particles morphology was studied using the EVO LS 10 LaB6 scanning electron microscopy (SEM) (Zeiss, Italy) with an acceleration voltage of 5 kV and a working distance of 5 mm. The samples were sputter coated under vacuum with a thin layer (10– 30 Å) of gold. Z-range = 63 ± 13 nm.
