# Supplementary material for: Mesenchymal stromal cells mediated delivery of photoactive nanoparticles inhibits osteosarcoma growth in vitro and in a murine in vivo ectopic model
Source: J Exp Clin Cancer Res. 2020 Feb 22;39:40. doi: 10.1186/s13046-020-01548-4 (PMC7036176; doi:10.1186/s13046-020-01548-4)
Supplement: Supplementary file 4 — Additional file 4: Figure 2S. 3D model characterization. Diameter measures (mean ± SD, n = 6) from day1 to day15 of spheroids prepared with the 3 OS cells lines (a) and representative images of spheroids prepared with 104 OS cells (b). Table 2S. Measures of 3D model morphological properties. Diameter and roughness coefficient measures from brightfield images of spheroids obtained from different OS cell lines after 3 days of culture in low attachment 96-well plate. [file 13046_2020_1548_MOESM4_ESM.pdf]

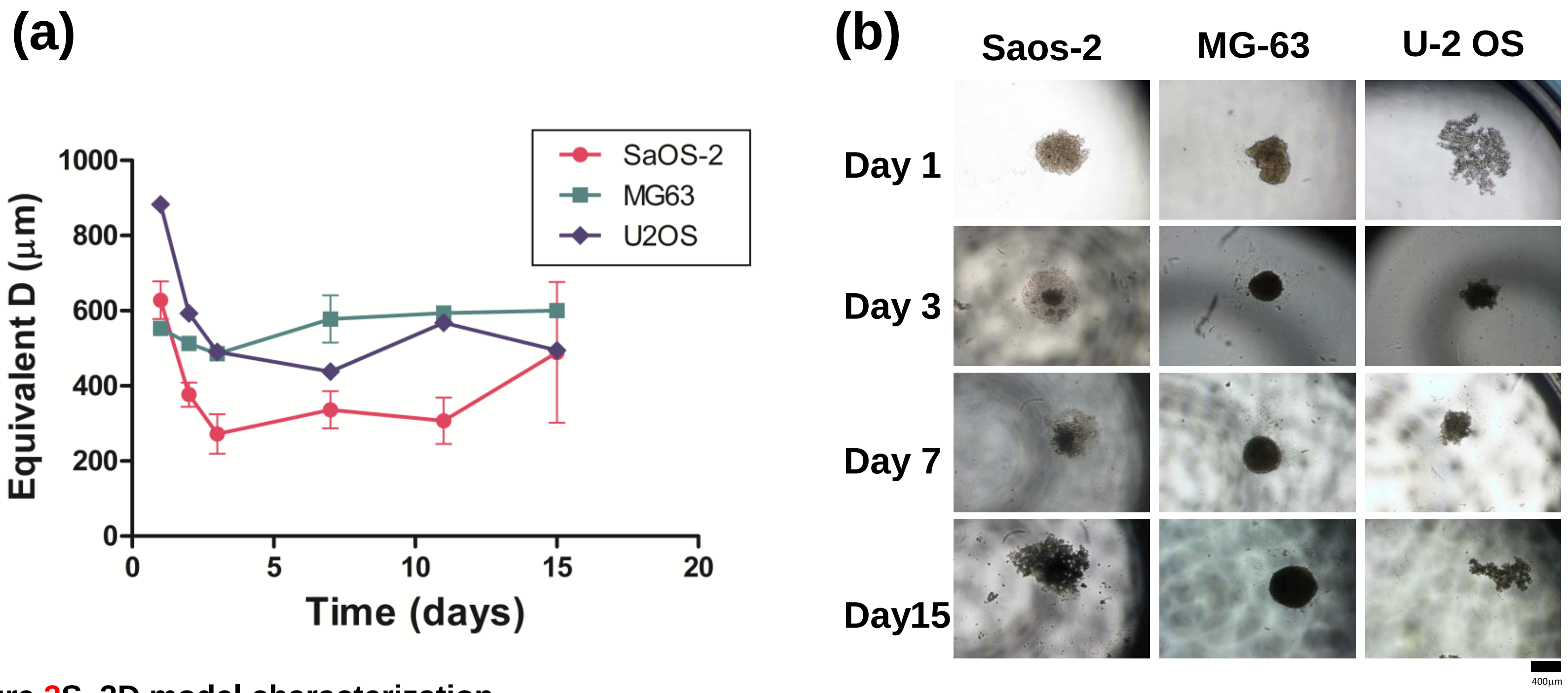

Figure 2S. 3D model characterization
Diameter ~~measures~~ (mean ± SD, n=6) from day1 to day15 of spheroids prepared with the 3 OS cells lines **(a)** and representative images of spheroids prepared with 10^4 OS cells (scale bar=400µm)**(b).**

Table 4S. Measures of 3D model morphological properties

	Saos-2			MG-63			U-2 OS		
	**Mean**	**CV%**	**N**	**Mean**	**CV%**	**N**	**Mean**	**CV%**	**N**
**Diameter (µm)**	272	19	6	485	9	6	490	10	6
**Roughness (perfect sphere=1)**	1,57	15	6	1,23	17	6	1,92	25	6

Diameter and roughness coefficient ~~measures~~ from brightfield images of spheroids obtained from different OS cell lines after 3 days of culture in low attachment 96-well plate.
