# Supplementary material for: Mesenchymal stromal cells mediated delivery of photoactive nanoparticles inhibits osteosarcoma growth in vitro and in a murine in vivo ectopic model
Source: J Exp Clin Cancer Res. 2020 Feb 22;39:40. doi: 10.1186/s13046-020-01548-4 (PMC7036176; doi:10.1186/s13046-020-01548-4)
Supplement: Supplementary file 5 — Additional file 5: Figure 3S. FNPs retention inside multicellular spheroids. Representative images of spheroids composed by MG-63 and AlPcS4@FNPs loaded MSC in 1:1 ratio, after 3 and 8 days of culture, showing the green fluorescent emission of AlPcS4@FNPs superimposed on the brightfield images of the whole spheroids (a). Quantification of fluorescence intensity inside the spheroids (n = 8) at the indicated timepoints; the spheroids’ perimeters traced on brightfield images were superimposed to corresponding fluorescent images to select a ROI and obtain the total intensity of green channel in the selected area (b). [file 13046_2020_1548_MOESM5_ESM.pdf]

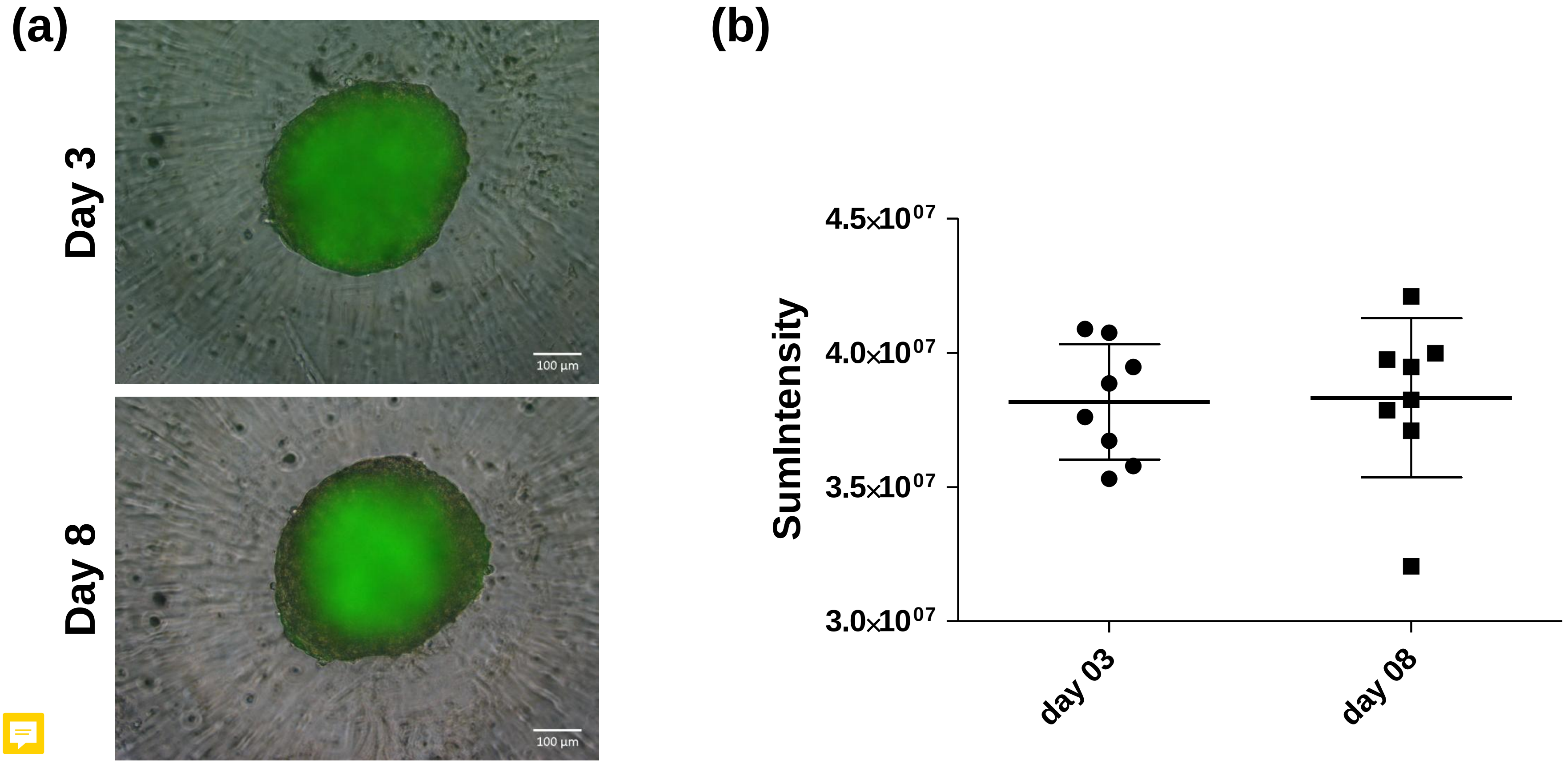

Figure 3S. FNPs retention inside multicellular spheroids.
Representative images of spheroids composed by MG-63 and $AlPcS_4$@FNPs loaded MSC in 1:1 ratio, after 3 and 8 days of culture, showing the green fluorescent emission of $AlPcS_4$@FNPs superimposed on the brightfield images of the whole spheroids **(a).** Quantification of fluorescence intensity inside the spheroids (n=8) at the indicated timepoints; the spheroids' perimeters traced on brightfield images were superimposed to corresponding fluorescent images to select a ROI and obtain the total intensity of green channel in the selected area **(b).**
