# Supplementary material for: Mesenchymal stromal cells mediated delivery of photoactive nanoparticles inhibits osteosarcoma growth in vitro and in a murine in vivo ectopic model
Source: J Exp Clin Cancer Res. 2020 Feb 22;39:40. doi: 10.1186/s13046-020-01548-4 (PMC7036176; doi:10.1186/s13046-020-01548-4)
Supplement: Supplementary file 6 — Additional file 6: Figure 4S. Survival rate tested in 5 MSC lines isolated from patients. Quantification of vitality by ATP assay (Cell Titer Glo 3D) 24 h after 10 min PDT in 5 MSCs cell lines loaded with 90μg/ml AlPcS4@NPs in co-culture with MG-63 cells in ratio 1:1. [file 13046_2020_1548_MOESM6_ESM.pdf]

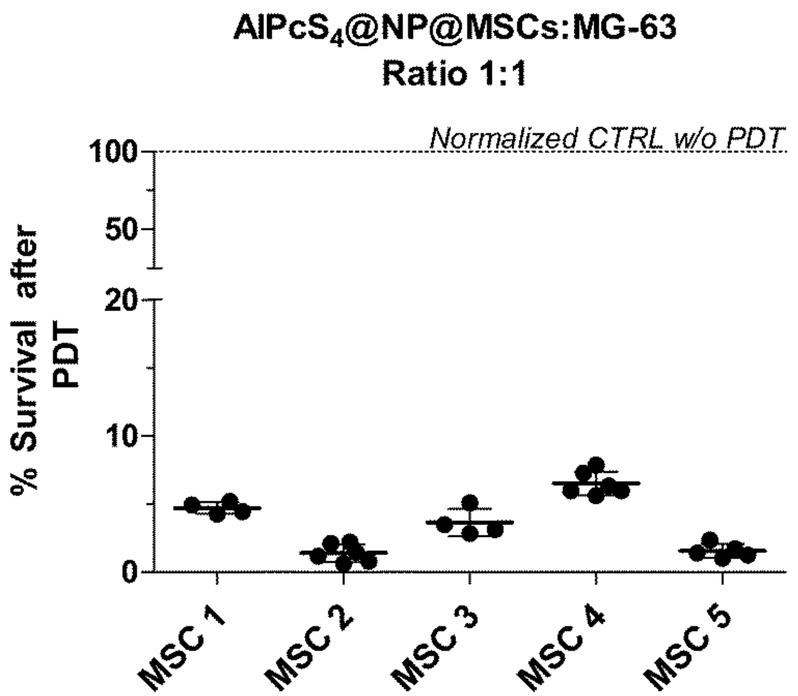

Figure 4S. Survival rate tested in 5 MSC lines isolated from patients.
Quantification of vitality by ATP assay (Cell Titer Glo 3D) 24h after 10 minutes PDT in 5 MSCs cell lines loaded with 90ug/ml $AlPcS_4$@NPs in co-culture with MG-63 cells in ratio 1:1.
