# Supplementary material for: Mesenchymal stromal cells mediated delivery of photoactive nanoparticles inhibits osteosarcoma growth in vitro and in a murine in vivo ectopic model
Source: J Exp Clin Cancer Res. 2020 Feb 22;39:40. doi: 10.1186/s13046-020-01548-4 (PMC7036176; doi:10.1186/s13046-020-01548-4)
Supplement: Supplementary file 7 — Additional file 7: Figure 5S. Full depth analysis of resin embedded spheroids. Toluidine blue stained semi-thin slices of one multicellular spheroid composed by AlPcS4@NPs loaded MSCs and MG-63 in a 1:7 ratio after 10 min irradiation (scale bar = 100 μm). Asterisks mark the areas where intact cells were observed. Slices correspond to different axial planes during cutting session as schematically explained in the first row. The location of each slice on the vertical axis depicted in the cartoon is approximated and arbitrary, since the orientation of the spheroids during cutting cannot be correlated with the orientation of the spheroid during PDT treatment. A central slice of a not irradiated spheroid is showed as positive control for the staining (blue frame). [file 13046_2020_1548_MOESM7_ESM.pdf]

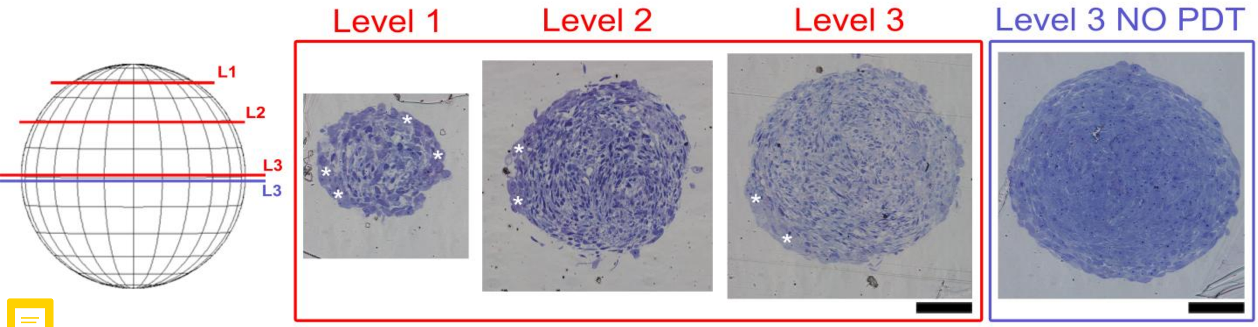

~~Figre~~ 5S. Full depth analysis of resin embedded spheroids

Toluidine blue stained semi-thin slices of one multicellular spheroid composed by $AlPcS_4$@NPs loaded MSCs and MG-63 in a 1:7 ratio after 10 min irradiation (scale bar= 100μm). Asterisks mark the areas where intact cells were observed. Slices correspond to different axial planes during cutting session as schematically explained in the first row. The location of each slice on the vertical axis depicted in the cartoon is approximated and arbitrary, since the orientation of the spheroids during cutting cannot be correlated with the orientation of the spheroid during PDT treatment. A central slice of a not irradiated spheroid is showed as positive control for the staining (blue frame).
