# Supplementary material for: Mesenchymal stromal cells mediated delivery of photoactive nanoparticles inhibits osteosarcoma growth in vitro and in a murine in vivo ectopic model
Source: J Exp Clin Cancer Res. 2020 Feb 22;39:40. doi: 10.1186/s13046-020-01548-4 (PMC7036176; doi:10.1186/s13046-020-01548-4)
Supplement: Supplementary file 8 — Additional file 8: Figure 6S. Representative imaging of photodynamic therapy effect on tumor bearing mice. Representative images of photoirradiated tumor on mouse left flank. Characteristic burn/scab formation was observed 24 h after second treatment in mice injected with AlPcS4@FNPs alone (white arrowhead). [file 13046_2020_1548_MOESM8_ESM.pdf]

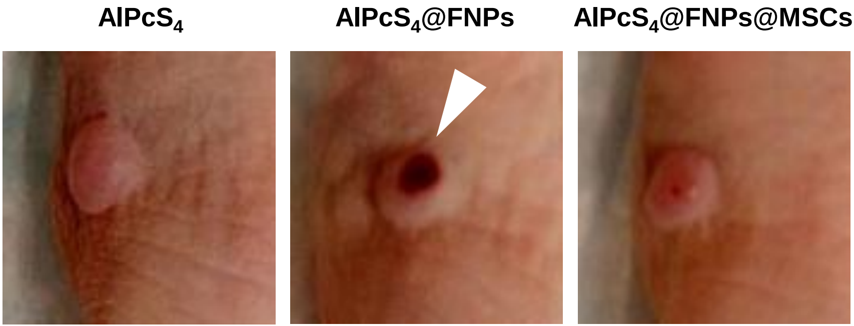

[illegible]igure 6S. Representative imaging of photodynamic therapy effect on tumor bearing mice

Representative images of photoirradiated tumor on mouse left flank. Characteristic burn/scab formation was observed 24h after second treatment in mice injected with $AlPcS_4$@FNPs NPs alone (white arrowhead).
